# Analysis of the Human Protein Atlas Image Classification competition

**DOI:** 10.1038/s41592-019-0658-6

**Published:** 2019-11-28

**Authors:** Wei Ouyang, Casper F. Winsnes, Martin Hjelmare, Anthony J. Cesnik, Lovisa Åkesson, Hao Xu, Devin P. Sullivan, Shubin Dai, Jun Lan, Park Jinmo, Shaikat M. Galib, Christof Henkel, Kevin Hwang, Dmytro Poplavskiy, Bojan Tunguz, Russel D. Wolfinger, Yinzheng Gu, Chuanpeng Li, Jinbin Xie, Dmitry Buslov, Sergei Fironov, Alexander Kiselev, Dmytro Panchenko, Xuan Cao, Runmin Wei, Yuanhao Wu, Xun Zhu, Kuan-Lun Tseng, Zhifeng Gao, Cheng Ju, Xiaohan Yi, Hongdong Zheng, Constantin Kappel, Emma Lundberg

**Affiliations:** 1 Science for Life Laboratory, School of Engineering Sciences in Chemistry, Biotechnology and Health, KTH–Royal Institute of Technology, Stockholm, Sweden; 2 Department of Genetics, Stanford University, Stanford, CA USA; 3 Chan Zuckerberg Biohub, San Francisco, CA USA; 4 Changsha, China; 5 Winning Health Technology Group Co., Ltd., Shanghai, China; 6 Seoul, Republic of South Korea; 7 Missouri University of Science and Technology, Rolla, MO USA; 8 Khumbu.ai, Munich, Germany; 9 Qualcomm, Inc., Cupertino, CA USA; 10 Brisbane, Queensland Australia; 11 H2O.ai, Greencastle, IN USA; 12 SAS Institute, Inc., Cary, NC USA; 13 Jilian Technology Group (Video++), Shanghai, China; 14 SAP, Moscow, Russian Federation; 15 BDO Unicon, Saint Petersburg, Russian Federation; 16 Ivanovo, Russian Federation; 17 Kharkiv National University of Radioelectronics, Kharkiv, Ukraine; 18 Santa Clara, CA USA; 19 UT MD Anderson Cancer Center, Houston, TX USA; 20 Shanghai, China; 21 University of Hawaii Cancer Center, Honolulu, HI USA; 22 Taipei, Republic of China; 23 Microsoft Research, Beijing, China; 24 University of California Berkeley, Berkeley, CA USA; 25 Beijing, China; 26 Peking University, Beijing, China; 27 Leica Microsystems, Mannheim, Germany

**Keywords:** Organelles, Machine learning, Image processing

## Abstract

Pinpointing subcellular protein localizations from microscopy images is easy to the trained eye, but challenging to automate. Based on the Human Protein Atlas image collection, we held a competition to identify deep learning solutions to solve this task. Challenges included training on highly imbalanced classes and predicting multiple labels per image. Over 3 months, 2,172 teams participated. Despite convergence on popular networks and training techniques, there was considerable variety among the solutions. Participants applied strategies for modifying neural networks and loss functions, augmenting data and using pretrained networks. The winning models far outperformed our previous effort at multi-label classification of protein localization patterns by ~20%. These models can be used as classifiers to annotate new images, feature extractors to measure pattern similarity or pretrained networks for a wide range of biological applications.

## Abstract (web summary)

The 2018 Human Protein Atlas Image Classification competition sought to improve automated classification of protein subcellular localizations from fluorescence images. The winning strategies involved innovative deep learning approaches for multi-label classification.

## Main

Advancement in high-throughput microscopy has propelled the generation of massive amounts of biological imaging data^1^. These data can offer valuable insights into cellular processes and biological systems, but only if we conquer the challenges of processing these voluminous datasets. The Human Protein Atlas (HPA) is a project that faces these challenges and opportunities. Through a systematic antibody-based approach, millions of fluorescence microscopy images have been generated to map the expression of the human proteome^2^. Compartmentalization is an important mechanism to allow multiple biological reactions to occur in parallel. In cells, these compartments are called organelles, and knowledge of which compartments our proteins reside in would greatly increase our understanding of human biology. The HPA Cell Atlas aims to map the subcellular distribution of the human proteome with confocal microscopy^3^. The current version (HPAv19) of the database comprises image data for 12,390 proteins (www.proteinatlas.org).

We have previously demonstrated an astounding degree of cellular complexity; as many as half of all human proteins are localized to multiple cellular compartments^3^, and many proteins show single-cell variability^4^. Unbiased analysis of subcellular protein localizations from our images has greatly enriched our vocabulary for describing cellular systems. This analysis was first performed manually^3^, and we have since integrated the labor-intensive annotation tasks into a mainstream video game^5^, which produced tens of millions of human annotations. These annotations were particularly successful at the challenging task of identifying mixed patterns of protein localizations, a task called multi-label classification^6^. Previously, we developed a machine learning model, called Loc-CAT, capable of classifying mixed patterns in images of cell types of different morphology^5^. However, the performance measured with macro F1 score (defined in Methods) was yet substantially lower (0.47) than that of human experts (0.71).

The emerging field of deep learning^7^ has powered many successful real-life applications, including image recognition^8^, gaming^9^ and autonomous cars^10^. Deep neural networks, particularly convolutional neural networks (CNNs)^8^, have been widely applied to perform computer vision tasks such as image classification^11,12^ and segmentation^13^. Compared to Loc-CAT^5^, which uses hand-crafted features as inputs, CNNs typically take raw images as inputs and learn hierarchical feature representations in an end-to-end fashion. This allows the model to better abstract cellular localization patterns and scale efficiently with data size^14^. CNNs are increasingly used for biological image analysis^15–17^ including multi-label classification for yeast protein localization^18^. Over recent years, a collection of successful neural network architectures, such as Resnet^19^, Inception^12^ and Densenet^20^, and different training techniques, such as Dropout^21^, Batch Normalization^22^, Focal Loss^23^, Cyclical learning rates^24^ and AutoAugment^25^, have been developed. Software libraries, such as PyTorch^26^ and Tensorflow^27^, can be easily implemented and applied to a wide range of applications. Automated machine learning^28^(AutoML) techniques such as hyperparameter optimization^29^, meta-learning^30^ and neural architecture search^31^ make the model development easier and accessible even for nonexperts.

Finding the best solution for classifying protein localizations within HPA Cell Atlas Images involves performing searches of parameters and hyperparameters over an enormous solution space. Crowd-sourced competitions are commonly used for large-scale solution searches. One such success is ILSVRC^32^, which provides the ImageNet dataset^33^ that is widely recognized as powering the current advancements in deep learning. Competitions for cellular image analysis have also been successfully introduced; for example, for classification^34^ and cell-tracking^35^. Popular online platforms such as Kaggle, Innocentive and DREAM allow publishing datasets and hosting competitions typically focused on benchmarking methods for fundamental research problems (for example, DREAM) or crowdsourcing solutions for real-life applications, often motivated by considerable prize money (for example, Kaggle).

Here, we present the design and results from our ‘Human Protein Atlas Image Classification’ competition, hosted by Kaggle. In contrast to typical image classification tasks that predict one label per image, our dataset requires classification of multiple labels per image (the multi-label problem^6^). There is also a great class imbalance in the dataset, making the classification task harder (the class imbalance problem^36^). During a 3-month period, 2,172 teams provided a total of 55,213 submissions, nearly all based on deep learning. The top-ranking solutions were awarded with a cash prize (Table 1, first place, US$14,000; second place, US$10,000; third place, US$8,000; fourth place: US$5,000). The models far outperformed our previous effort at protein localization classification, and there was considerable variety among the solutions with some convergence on popular networks and training techniques. Different strategies for adapting neural networks and loss functions, augmenting data and using pretrained networks were successfully applied by the winning teams. Here, we present the competition design, statistical analyses of the solutions and visualizations of the winning models to shed light on the considerations for designing multi-label pattern classification algorithms and potential applications of the winning solutions.

**Table 1:** Models and their performance for top ranking and selected teams

Rank	Team Name	Member(s)	Score	Award (US$)
1	Team 1: bestfitting	D. Shubin	0.593	14,000
2	Team 2: WAIR	J. Lan	0.571	10,000
3	Team 3: pudae	P. Jinmo	0.570	8,000
4	Team 4: Wienerschnitzelgemeinschaft	S. Mahmood Galib, C. Henkel, K. Hwang, D. Poplavskiy, B. Tunguz, R. Wolfinger	0.567	5,000
5	Team 5: vpp	Y. Gu, C. Li, J. Xie	0.566	–
8	Team 8: One More Layer (Of Stacking)	D. Buslov, S. Fironov, A. Kiselev, D. Panchenko	0.563	–
10	Team 10: conv is all u need	X. Cao, R. Wei, Y. Wu, X. Zhu	0.557	–
16	Team 16: NTU_MiRA	K.-L. Tseng	0.553	–
39	Team 39: Random Walk	Z. Gao, C. Ju, X. Yi, H. Zheng	0.540	–

## Results

### Competition design and assessment metrics

**Fig. 1:**
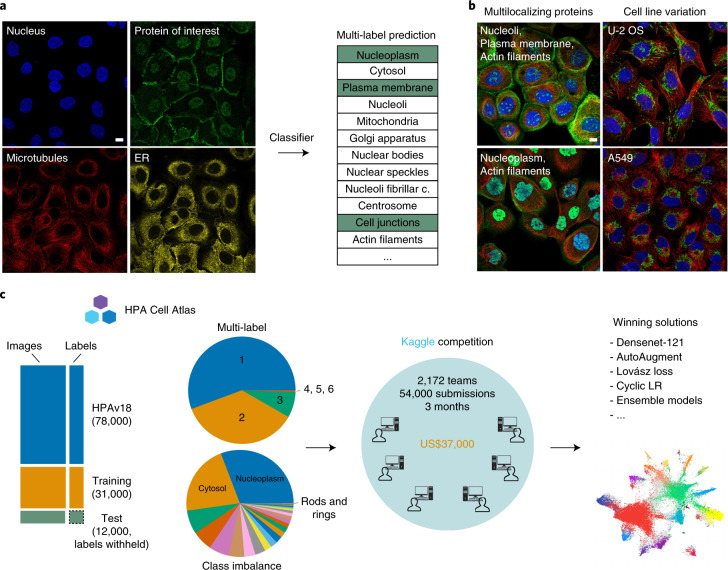
Overview of image dataset and challenge design. **a**, A typical HPA Cell Atlas image and the aim of the competition. Each image consists of four channels: the antibody-stained protein of interest (green) and three reference channels to outline the cell: microtubules (red), nucleus (blue) and endoplasmic reticulum (ER; yellow). The human cell comprises many compartments, here defined by 28 labels. The aim of the competition is to build classifiers to predict the localization pattern (often multiple labels) of the protein of interest. Scale bar, 10 μm. **b**, Sample images showing different protein or cell line expression patterns that make the pattern classification task challenging. Proteins localizing to multiple compartments are exemplified by Septin 7 in A-431 cells (left, top), and PRAME family member 12 in A-431 cells (left, bottom). Stainings of mitochondria (TOMM70, Translocase of outer mitochondrial membrane 70, in U-2 OS and CCR7, C-C motif chemokine receptor 7, in A459) show the morphological differences between cell lines (right, from top to bottom). Scale bar, 10 μm. PM, plasma membrane; Actin fil., actin filaments. **c**, Challenge overview: the HPA is a proteome-wide image collection detailing protein localization. This dataset is challenging to analyze automatically because of prevalent multi-label classifications (1–6 labels per image, upper pie chart) and high imbalance among the 28 different protein localization classes (lower pie chart). To find the best solution for these problems, we held a competition hosted by Kaggle. The challenge dataset consisted of 42,774 images with labels from expert annotations and was divided into a training set and test set before distributed to the Kaggle challenge participants with the labels of the test set withheld. We used a macro F1 score to assess the performance of these models. The competition produced winning solutions and different methods for multi-label image classification. LR, learning rate.

The aim of the competition was to develop computational models for the classification of protein subcellular localization patterns in confocal microscope images from the HPA Cell Atlas (Fig. 1). We prepared a dataset of 42,774 nonpublic images and allowed participants to use any external data, including the ~78,000 images that are publicly available on the HPA Cell Atlas (HPAv18). Each image contained multiple cells and had four channels marking the protein of interest and cell outlines (Fig. 1a).

**Fig. 2:**
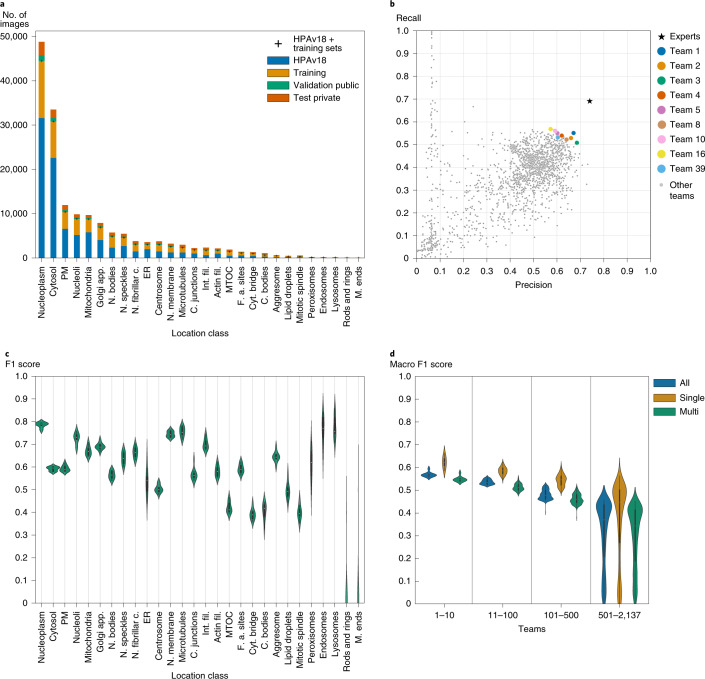
Competition results. **a**, Image numbers of each localization class for HPAv18, training, validation_public and test_private dataset. PM, plasma membrane; Golgi app., Golgi apparatus; N. bodies, nuclear bodies; N. speckles, nuclear speckles; N. fibrillar c., nucleolar fibrillar center; ER, endoplasmic reticulum; N. membrane, nuclear membrane; C. junctions, cell junctions; Int. fil., intermediate filaments; Actin fil., actin filaments; MTOC, microtubule organizing center; F. a. sites, focal adhesion sites; Cyt. bridge, cytokinetic bridge; C. bodies, cytoplasmic bodies; M. ends, mitochondrial ends. **b**, Precision-recall values for the experts, selected teams (including the top four winning teams) and all other teams. **c**, Statistics on the macro F1 scores of different teams and their performance on different classes. Score distributions for the different label classes with the classes sorted according to sample size (high to the left, low to the right). *n* = 10 teams for each violin. The minimum (min), mean, percentile (P) and maximum (max) values can be found in Supplementary Table 9. **d**, Statistics on the macro F1 scores of different teams and their performance, binned into groups based on their ranking on the leaderboard. The top 10, 11–100, 101–500 and the remaining teams, respectively. The scores for single localized, multi-localized and all proteins are shown separately. *n* = 10 teams for violins with teams 1–10, *n* = 90 teams for violins with teams 11–100, *n* = 400 teams for violins with teams 101–500 and *n* = 1,637 teams for violins with teams 501–2,137. The minimum, mean, percentile and maximum values can be found in Supplementary Table 9.

We designed the competition to address the two main challenges of developing computational models for this purpose, namely the class imbalance and multi-label problems (Fig. 1). The first difficulty arises from the highly imbalanced frequencies of the 28 localization classes (Fig. 2a and Supplementary Table 1). The most common label in the training set was ‘nucleoplasm’ (12,885 images in training and 31,590 images in HPAv18), while the rarest label was ‘rods and rings’ (11 images in training and 42 images in HPAv18, see Supplementary Table 2). The second difficulty, the multi-label problem, stems from the need to potentially assign multiple labels to each image. Each image has been assigned 1–6 such labels during the standardized annotation pipeline^3^ (Fig. 1c, Methods). In total, the dataset contains 577 unique combinations of labels. Beyond these main difficulties, different cellular morphologies of the 27 cell lines in our dataset add complexity (Supplementary Table 3).

Typically, learning algorithms perform well on common classes but perform poorly on rare classes. To encourage an equally distributed classification performance among all the 28 classes, we chose to assess model performance with a macro F1 score (range 0–1). This score gives importance to both precision and sensitivity (recall), and is calculated for each class before averaged over the 28 classes. To earn a high score in this competition, a model thus needs to pay special attention to rare classes. During the competition, teams could see their score and rank on a public leaderboard, which was computed from a subset of test images (‘validation_public’, see Fig. 2a). At the end of the competition, another subset of test images (‘test_private’, see Fig. 2a) was used to produce the final ranking on the private leaderboard (Methods). During the competition, ~148 images of rare classes were mistakenly included from the published Cell Atlas Images; this was fixed during the competition by excluding the leaked images from the ‘test_private’ set (full disclosure in Methods). Since the participants were notified and the final evaluation was performed with the ‘test_private’ set, the overall impact of the leakage on the results of this paper is minimal.

### Participation and performance

After the final ranking, we awarded the top four teams based on their rank on the private leaderboard (Table 1). The winning team generated models with macro F1 scores ranging from ~0.56–0.59, >20% better than Loc-CAT and the citizen science results. We invited nine teams among the top 40 to participate in this study.

When analyzing the final submission for all the teams (2,137 submissions), we found that the top teams were marginally better than most other teams. Both the precision and recall of experts are substantially higher than the top teams. Figure 2b shows the precision-recall for all the teams and the experts’ score (on a similar dataset; precision, 0.74 and recall, 0.69) from our previous work^5^. We computed macro F1 scores for groups of teams binned based on their ranking on the private leaderboard (Fig. 2d). The scores for teams with higher ranks varied less, which indicates it is harder to improve further for higher ranking models. The scores for single label images are significantly higher (Methods, *P* < 1.08 × 10^−5^, two-sample Kolmogorov–Smirnov test) than for multi-label images for all groups, emphasizing the difficulty of classifying images with mixed localization patterns.

To understand the impact of class imbalance on the performance of the models, we computed the class-wise score distribution for the top ten teams. Figure 2c and Supplementary Table 4 show that the averaged F1 score, precision and recall for each class varied more when the class had fewer samples in the training dataset; in particular, most models struggled with the two rarest classes (rods and rings, microtubule ends). The difficulty of identifying different localization patterns in the images also played a role in performance for each class. For example, the average F1 score was higher for aggresomes than plasma membrane, despite a lower number of training images (322 and 3,777, respectively), because the aggresome is visually distinct while the plasma membrane is often confused with the cytosol. The performance correlated better with the sample number for cell lines than for classes (Supplementary Fig. 1), likely because the assessment metric (macro F1) encouraged participants to develop models that are equal in performance for the different classes, rather than the different cell lines. A confusion matrix (Supplementary Fig. 2) for single-class samples based on the winning model shows that the endoplasmic reticulum and peroxisomes were confused with cytosol, and that nuclear speckles were mistakenly classified as nucleoplasm. The patterns are consistent across cell lines, despite morphological differences, showing that the model generalizes well (Supplementary Fig. 2).

### Strategies used by the top-ranking solutions

To compare the underlying structures of the solutions, we invited the top 200 teams to fill out a survey on the methodology used, which was answered by 56 teams. Notably, all teams but one used deep learning models. For the neural network architectures, 44 of the 56 teams used variations of Resnet, Densenet or Inception as backbone architecture, as they are known to be effective for image recognition tasks^12,19,20^. To address the multi-label problem, most teams (34 of 56) used binary cross entropy. Many teams handled class imbalance by applying class weights or by using focal loss^23^ to train the models, and employed multi-label stratification^37^ to generate validation datasets. Most teams used the public HPAv18 dataset for training; adding these ~78,000 annotated images led to a substantial boost in image classification scores (for example, from 0.510 to 0.552 for Team 1, see Supplementary Tables 5b and 6), mostly due to the increase of rare class images. Augmentation strategies such as random cropping, rotation and flipping were commonly used (Supplementary Notes and Supplementary Table 5).

To understand how these different strategies contribute to performance, we collected detailed information about the solutions and intermediate experiments from nine selected teams (Table 1, Supplementary Table 5 and Supplementary Figs. 3–11). Among these teams, different strategies were applied to different aspects of image analysis. For example, Team 1 used an optimized single neural network and a combined loss function with a Lovász loss^38^ term, Team 2 focused on data preprocessing, Team 3 used automatic data augmentation and Team 4 ensembled a large number of models.

We found that different teams often drew the same conclusion on a number of strategies, despite the fact that they mostly worked independently. Team 3 employed an automated augmentation strategy search algorithm (AutoAugment^25^), which resulted in an improvement of the macro F1 score from 0.477 to 0.499. In addition to training time augmentations, test time augmentations were also shown to be effective by several teams. Both Team 1 and 5 found DenseNet^20^ to be more effective than Resnet^19^ in terms of neural network architecture. In terms of network size, Teams 1 and 4 found medium-sized networks (for example, Densenet 121) to be better than larger ones (for example, Densenet 169). Several teams, including 1, 2, 3 and 16, found that scores can be improved by using a larger image size (for example, 1,024 × 1,024 pixels). Teams 4 and 10 even applied models that work on multi-scale. Techniques such as model ensembling and stacking were used to push the performance beyond single models. By ensembling single models from the top three teams, we obtained an even better model (macro F1 = 0.575, Supplementary Table 7).

To facilitate the reuse of these solutions, we built a model zoo (https://modelzoo.cellprofiling.org) to host the source code and trained models from the selected teams. These can be used as pretrained models^39^ to reduce the training time and training data size for constructing new models for biological image analysis.

### Assessing the biological relevance of the winning model with class activation maps (CAMs)

**Fig. 3:**
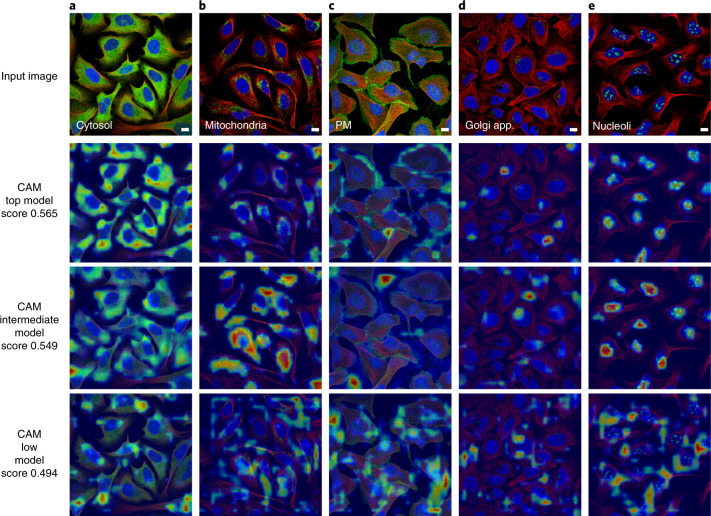
Visualization of model spatial attention. CAMs for three different models, the top-scoring model (from Team 1), an intermediate-scoring model (from Team 3) and a low-scoring model (from Team 1). Scale bars, 10 μm. **a**, For the cytosolic protein Methenyltetrahydrofolate synthetase, the CAMs for all three models highlight relevant cellular regions. **b**, The CAMs for the mitochondrial protein Prohibitin 2 show a progressively worse overlap with the mitochondrial staining following the model accuracy score. **c**, The plasma membrane staining of Catenin beta 1 overlaps well with the CAM for the top model, but not for the intermediate and lower scoring models. **d**, The CAMs for Golgi reassembly stacking protein 1, which is localized to the Golgi apparatus, show attention of correct size for all three models, but none of the models focused on all cells in the image. **e**, The nucleolar staining pattern of UTP6 small subunit processome component, is captured well by the CAMs for the top and intermediate models in the nuclear region of the cell.

Understanding whether a prediction for a given image is based on biologically relevant information is difficult due to the poor interpretability of neural networks. However, newly developed visualization techniques, such as class activation mapping^40^, allow us to peer into the spatial attention of these models to ensure that the classification is based on biologically meaningful information. A model that fails to focus on the biologically relevant regions of a cell generally indicates lower performance. In Fig. 3, we compared the best, an intermediate, and a low scoring model (Methods) by generating CAMs from ‘easy’ (cytosol and nucleoli) and ‘hard’ patterns (mitochondria, plasma membrane, Golgi apparatus). For the easy patterns, the CAMs visualize biologically relevant regions for the top and intermediate model. For the hard patterns, we see more diversity between the models and images.

CAMs can also be used to identify when the score fails to reflect biological information. The final solution by Team 1 is an ensemble of two models (Supplementary Fig. 3): a classification model and a metric learning model^41^ that was trained with antibody identifiers from HPAv18 (Supplementary Notes, Supplementary Fig. 12 and Supplementary Table 5). The metric learning part boosted the score by ~4% (macro F1 of 0.565 with the classification part alone to 0.593). However, we found that the metric learning model mainly gained performance by pairing images in the test set to those acquired from the same sample in HPAv18 (Methods). With the help of CAMs, we found that the visual attention pattern varied from protein to protein and often focused on biologically meaningless features (Supplementary Fig. 13). Presumably, the metric learning model works by exploiting ‘batch effects’ in the dataset through so called ‘hidden variables’, a pitfall in machine learning^40^. Nevertheless, the classification model without metric learning retained the record of single model performance (macro F1 = 0.565, Supplementary Table 5) with verified performance in a five-fold cross-validation experiment (Supplementary Table 8).

### Visualizing image feature representations of subcellular protein distributions

**Fig. 4:**
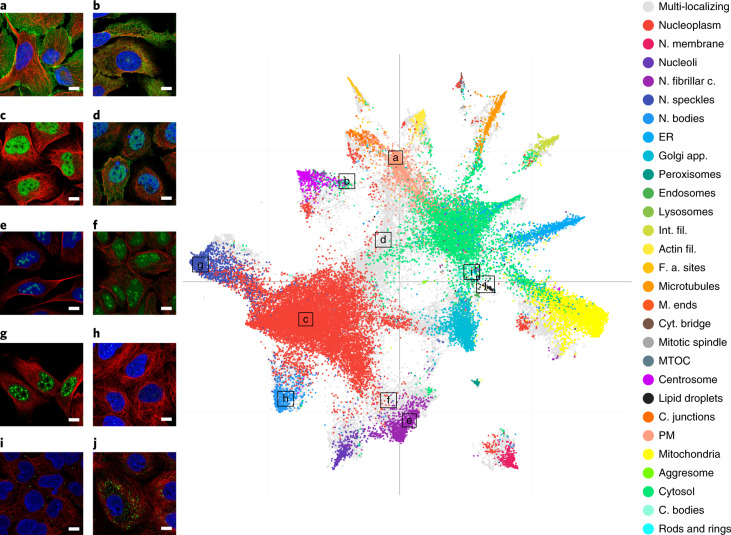
Visualization of learned features. UMAP visualization of the features learned by the best scoring model from Team 1 with a few corresponding original images highlighted. Single location images are colored according to location, while gray data points belong to multi-localizing proteins. Abbreviations as in Fig. 2. Scale bars, 10 μm. **a**, Catenin beta 1 is localized to the plasma membrane and also appears in the plasma membrane protein cluster. **b**, Although trained on the manual labels, this type of unbiased analysis provides a tool to identify misclassified patterns or subtle pattern variations. The protein suppressor of cytokine signaling 3 with the label ‘cytosol’ is found among the centrosome/microtubule organizing center (MTOC) cluster. After visual inspection, we can indeed identify an enrichment of this protein around the MTOC in addition to the cytoplasm in some cells. **c**, RUNX1 translocation partner 1 is localized to the nucleoplasm and appears in the nucleoplasmic protein cluster. **d**, Utrophin is localized to both the plasma membrane and nucleoplasm and appears between these two respective clusters. **e**, EBNA1 binding protein 2 is localized to nucleoli and appears in the nucleoli cluster. **f**, L3MBTL3 histone methyl-lysine binding protein is localized to both the nucleoli and nucleus, and appears between these two respective clusters. **g**,**h**, Heterochromatin protein 1 binding protein 3 is localized to nuclear speckles (**g**) and Centromere protein T (**h**) is localized to centromeres. Despite the pattern similarities of the two categories, they still appear in two distinct clusters. **i**,**j**, Enhancer of mRNA decapping 4 protein is localized to cytoplasmic bodies (**i**), generating a similar staining pattern as Perilipin 3, which is localized to lipid droplets (**j**). Despite the similarities of the two categories, they still appear in two distinct clusters.

To investigate the ability of a CNN to distinguish subcellular structures, we analyzed features extracted from the penultimate layer of the classification model from Team 1 (without the metric learning model). Figure 4 shows a uniform manifold approximation and projection for dimension reduction (UMAP)^42^ projection of these features. First, the features clearly distinguish the different subcellular locations. Nuclear sub-compartments cluster separately, such as the nucleoplasm (for example, RUNX1 translocation partner 1), nucleoli (for example, EBNA1 binding protein 2), nucleoli fibrillar center, nuclear bodies (for example, Centromere protein T) and nuclear speckles (for example, Heterochromatin protein 1 binding protein 3); these examples also illustrate how well fine structures are distinguished, such as the assignment of heterochromatin protein 1 binding protein to nuclear speckles and centromere protein T to nuclear bodies (that is, centromeres). Similarly, the proteins Enhancer of mRNA decapping 4 and Perilipin 3 are accurately predicted to localize to cytoplasmic bodies and lipid droplets. Although these cellular structures are small puncta in the cytosol, the model can reliably distinguish them.

Images that were assigned multiple localization labels (gray points in Fig. 4) are found between clusters of images representing the single locations. Examples include L3MBTL3 histone methyl-lysine binding protein, located in both the nucleus and nucleoli, and Utrophin, located in both the nucleus and plasma membrane. This indicates that the features learned by the CNN have the potential to be used as quantitative representations of mixed patterns.

A web application was developed and deployed with the ImJoy platform^43^, where users can interact with the UMAP and the images with associated metadata (https://tinyurl.com/y6nhf5bo). This provides a new way to explore large online resources, such as the HPA database.

## Discussion

The HPA image classification competition provides crowd-sourced solutions for the task of classifying protein subcellular localization patterns in fluorescence microscopy images. The participants were tasked with developing solutions to solve the multi-label classification problem on a dataset with high class imbalance. The participants tested a large number of techniques in a competitive setup, which led to the use of external datasets and methods not previously applied to multi-label classification.

A key design choice for the competition was to use macro F1 as the assessment metric. It successfully encouraged the participants to optimize their solution to handle the label class imbalance in our dataset and yielded roughly similar performances among the classes except the rarest two. During the challenge, many strategies were implemented, such as adapting Lovász loss (designed for segmentation) to multi-label classification, developing the metric learning model and testing a large number of new models and training techniques. As a result, the performance of the winning models are substantially better than our previous Loc-CAT model, but not yet at the level of human experts. More comparative experiments are required to better evaluate the gap between our models with human experts.

Despite the superior performance of deep learning methods, there are limitations including hallucination and generalization problems^44^. It has also been reported that machine learning algorithms can easily pick up unintentional variations (for example, in biomedical image classification^45^). We speculate that the metric learning model used these unintended hidden variables^46^(for example, background noise) to match the test images with HPAv18 and boost the performance. Since this type of problem cannot be reflected by the evaluation metric, special attention is required for future competition organizers to prevent this type of exploitation.

We envision that the pretrained models provided in our model zoo will be useful in the context of transfer learning, a popular method that dramatically reduces training time and improves the generalization of learning models^39^. As common practice, models pretrained with ImageNet^32,33^ are used even when applied to microscopy images of cells^47^, showing the robustness of transfer learning. However, it has also been shown that the generalization of the ImageNet features is questionable, especially for fine-grained classification tasks^48^. Compared to the classes in ImageNet (for example, house and plane), the patterns in cells (for example, mitochondria and centrosome) are much finer grained. Thus, we foresee that applications involving biological images will adopt the HPA classification models trained with a large number of cell microscopy images instead of using ImageNet. Furthermore, our HPA competition dataset can be used as a benchmark dataset similar to ImageNet for developing new algorithms. For example, it may aid in designing models that handle high class imbalance or advancing cell mapping research by developing models for unsupervised analysis of subcellular protein patterns in single cells.

Although the top model could not reach expert level performance, it still opens up avenues for advancing cell biology. The use of high-dimensional features (as in the UMAP) instead of discrete labels constitutes an attractive approach for building systems level representations of cells harboring information about both single and multi-localizing proteins and serves a basis for quantitative integration of spatial information with other ‘omics data.

## Methods

### Competition and prizes

This paper describes the outcome of the HPA Image Classification competition at Kaggle (https://www.kaggle.com/c/human-protein-atlas-image-classification/), which was active from 3 October, 2018 to 10 January, 2019. The top-ranking solutions were awarded with a cash prize (Table 1, first place, US$14,000; second place, US$10,000; third place, US$8,000 and fourth place, US$5,000).

### Image generation in the HPA Cell Atlas

In the HPA Cell Atlas, each target protein is imaged in three different cell lines selected based on messenger RNA expression data from a large panel of cell lines. A standard immunostaining protocol is applied in a 96-well format to stain the target protein^49^ (https://www.protocols.io/view/standardized-immunohistochemical-staining-used-in-yj8furw). To facilitate the downstream annotation process, reference markers for the nucleus, microtubules and endoplasmic reticulum are also stained. Confocal microscopes (63× oil immersion) are used to image each sample in the 96-well plate, and multiple images are typically acquired for each well. The four-color images (image size 2,048 × 2,048; 16-bit, pixel size 0.08 µm or 3,072 × 3,072; 16-bit, 0.08 µm) are uploaded to our laboratory information management system, where automatic quality checks are applied to pass only in-focus images with high contrast and good staining intensity. Manual annotation of the observed localization pattern is performed by applying one or more labels to the images (typically two to six) from each sample (same antibody, cell line and sample preparation date^3^). This dataset contains a mixture of images annotated by two types of workflow: (1) experts annotate the image, another expert curates it and (2) gamers from EVE Online^5^ annotate the images and the annotations are curated by experts, as described in (1). Typically, at least two images per antibody per cell line are selected to create the dataset. In HPAv18, 32 labels are used to describe cellular localization classes.

### Dataset assembly and quality control

The total dataset consisted of 42,774 images. The training set had 31,072 images, and the test set had 11,702 images. We provided all images both in high resolution (a mix of 2,048 × 2,048 and 3,072 × 3,072 pixels, TIFF 8-bit image files) and low resolution (512 × 512 pixels, PNG 8-bit grayscale files). Note that the PNG files were directly resized from TIFF images. The size inconsistency for the TIFF images were due to the variation of the actual size of acquired area from the sample originally, but they all shared the same pixel resolution. A total of 32 organelle classes from the HPA were merged into 28 classes for the competition (see Supplementary Table 1 for details of how the classes were merged and Supplementary Table 2 for the distribution of the classes within the training and test sets). Since the HPA Cell Atlas includes over 30 different cell lines, we sampled across 27 main cell lines, and have a roughly equal distribution across different cell lines. Sampling was done in groups consisting of each cell line and organelle combination to achieve this effect. Still, some groups were smaller than others, due to the imbalanced nature of the total HPA data, both regarding the class labels applied and the cell lines used in the experiment (see Supplementary Table 3 for the cell type image distribution in the training and test sets).

The test dataset images were collected first from nonpublished images generated within HPA. We excluded images from the same biological sample (with a specific protein and cell line) already represented in the test set from the training set to avoid information leakage. The training set images were collected from both public and nonpublic HPA Cell Atlas images.

For quality control, we applied further image analysis to get an acceptable quality of the images in the competition dataset. This allowed us to use nonpublished images from the HPA Cell Atlas, which are of mixed quality (high and low) compared to the high quality of the published images. The quality control was based on cell count and image contrast. The cell count was performed on the red (microtubules) channel images, mainly by applying a Gaussian filter and otsu thresholding with the scikit-image^50^ library and by removing objects smaller than 8,000 pixels. We required a minimum of five cells per image and excluded cells touching the image borders. A minimum contrast check was applied to the green channel (protein of interest) with the ‘is_low_contrast’ method from scikit-image. We set ‘fraction_threshold’ to 0.2 and ‘upper_percentile’ to 99.99. Low contrast images were discarded.

### Image-wise classification task

In this competition, the classification task is performed image-wise mostly because our current annotations are made at this level for each protein/antibody and cell line. We do not provide cell-wise labels, although it may yield better performance due to single-cell variations. However, we expect the impact to be minimal because only ~2% of proteins vary in their localization patterns between cells in images^3^ and because these images are assigned the labels for all patterns observed in the image. Future improvements along this direction may be targeted to these proteins show single-cell variations.

### Image resolutions and external data

We provide the entire image dataset in high and low resolutions, and we allowed the participants to explore the use of external data and pretrained models. We believe this is important, because it allowed the participants explore not only the model itself, but also different strategies to fetch external data, augment the data and take advantage of pretrained models, which had already been shown to be effective. We also believe it is important to obtain better performance under realistic constraints.

For the hidden variable problem raised from the metric learning model, prohibiting the use of external training data (and pretrained networks) may reduce the same type of risk. However, this may also prevent teams from achieving the desired outcome, since external data can improve performance, as we observed with HPAv18. A compromise may be to restrict the set of information allowed for training, such as only location labels from HPAv18 to avoid finding weak correlations with extra information such as antibody identifiers.

### Data leakage disclosure and fix during the competition

During the competition, participants notified us that there was a data leakage issue after comparing the public HPA Cell Atlas Images (HPAv18) with the test set images using similarity analysis (for example, perceptual image hashing). We identified that 148 out of 11,702 images in the test set (including ‘validation_public’ and ‘test_private’) were mistakenly included. We also noticed that all the leaked images contain rare class labels, and many of the leaked images were not identical to images in HPAv18 but highly similar, for example by coming from a different focal plane.

Shortly after the leakage was identified, most of the leaked images in ‘test_private’ (the final evaluation test set to generate the ranking on the private leaderboard) were removed from scoring, and the rest of the leaked images were swapped with unleaked images with the same labels from ‘validation_public’ to keep rare classes in both test sets. All participants were notified of the leak and the fix. Most teams detected leaks in their code and excluded those leaked images from their own validation dataset.

### Performance criteria

The F1 scores computed in Fig. 2 were computed from the following equation:

$${\mathrm{Precision}} = \frac{{T_{\mathrm{p}}}}{{T_{\mathrm{p}} + F_{\mathrm{p}}}}$$

$${\mathrm{Recall}} = \frac{{T_{\mathrm{p}}}}{{T_{\mathrm{p}} + F_{\mathrm{n}}}}$$

$${\mathrm{F1}} = 2 \times \frac{{{\mathrm{precision}} \times {\mathrm{recall}}}}{{{\mathrm{precision}} + {\mathrm{recall}}}}$$

where *T*_p_ denotes the number of images that are true positive, and *F*_n_, *F*_p_ denote the false negative and false positive, respectively.

The macro F1 score is computed from:

$${\mathrm{macro}}\ {\mathrm{F}}{1} = \frac{1}{n}\left( {\mathop {\sum }\limits_{i = 1}^n {\mathrm{F1}}} \right)$$

During the challenge, we used macro F1 to compute the score for public leaderboard rankings. Each team was allowed to select two final submissions at the end of the challenge. Macro F1 was used to compute the score on the private leaderboard (if there were two submissions for a team, we took the maximum score), and this was used as the main criterion to award the teams.

To analyze the score distributions of all teams, we took the one submission per team that gave the maximum score on the private leaderboard. For Fig. 2d we computed the score for all, single- and multi-localized classes with macro F1. Per-class F1 scores in Fig. 2c were computed with F1 for each class with no averaging. Scores shown in tables were rounded down.

### Public and private leaderboard

During the competition, teams were scored and ranked on a public leaderboard. To prevent overfitting to the public leaderboard, a subset (29%) of the test set was used for calculating the leaderboard scores, while the remaining part (71%) of the test set was preserved for the final evaluation. This is important because participants tend to optimize toward higher scores on the public leaderboard at the risk of overfitting to the test data.

Since the participants ran their own model and only submitted the predicted labels for the test set, it was mandatory for the top four winning teams to submit their models for further inspection.

### CAMs

We used Grad-CAM^40^ to produce the CAMs shown in Fig. 3 and Supplementary Fig. 13 from a chosen reference convolutional layer of the network under investigation. We generated CAMs for the following models in Fig. 3: Model 1 is densenet121_1024 from Team 1, Model 2 is inception-v3 from Team 3, Model 3 is densenet121_standard_no_crop and the metric learning model from Team 1. The architecture of these three models are shown in Supplementary Fig. 12. For generating CAMs, the reference convolutional layer of these models was set to Block3, Mixed_7c, Layer3 and Layer3, respectively. The generated CAMs were resized and overlaid on top of the corresponding input image. These models are also described in Supplementary Notes: experiment 18 in Supplementary Table 5b, experiment 8 in Supplementary Table 5f, experiment 30 in Supplementary Table 5b and model 5 in Supplementary Table 5a.

### Feature visualization with UMAP

For the feature visualization in Fig. 4, we used the 1,024-dimension feature from the ‘fc’ layer (as shown in Supplementary Fig. 12) of the densenet121_1024 model from Team 1. It was projected with UMAP to reduce the dimensionality from 1024 to 2. For generating the UMAP, the number of neighboring points, minimal point distance and number of components metric were set to 15, 0.1 and 2, respectively. The distance metric was set to Euclidean distance. We processed all the images in the training set and the entire HPAv18 dataset, and the generated 2D vectors were then plotted as a scatter plot. The data points are color coded are corresponding to their annotated location.

For the live HPA-UMAP (https://tinyurl.com/y6nhf5bo), we wrote the ImJoy plugin in Javascript and used Plotly.js to make the plot. Images shown when clicking the data points were pulled from the proteinatlas.org dynamically.

### Metric learning model results evaluation

The score boost for the metric learning is mostly because of identification of ‘batch effects’ defined as images derived from different regions of the exact same sample (antibody and cell line combination), and not mainly from improved performance for rare classes. In total, 935 images in the ‘test_private’ set has one other image from the same sample in HPAv18 and the metric learning model detects 647 (69.2%) of them. We found 270 images in the ‘test_private’ set where the classification model made wrong predictions, but were successfully corrected by the metric learning model. Among these images, 261 have at least one image from the same sample in HPAv18 and 34 of them belong to rare classes (rare class defined as containing fewer than 1,000 images in the dataset). However, there are 71 images from the ‘test_private’ set (including five rare images) that were correctly predicted by the classification model, but replaced into wrong labels by the metric learning model.

### Statistical analysis

The plotting and statistical analysis were performed with Python 3.6, NumPy, SciPy, scikit-learn, Pandas, seaborn and Matplotlib. To test the Macro F1 score difference between single label and multi-label images a two-sample Kolmogorov–Smirnov test was performed for significance testing. The test returns a two-tailed *P* value. The scores for single label images are significantly higher (*P* < 1.08 × 10^−5^) than for multi-label images for all groups. The test was done for each group of teams, 1–10: *n* = 10 teams (*P* < 1.08 × 10^−5^), 11–100: *n* = 90 teams (*P* < 3.96 × 10^−51^), 101–500: *n* = 400 teams (*P* < 5.22 × 10^−197^) and 501–2,137: *n* = 1,637 teams (*P* < 4.01 × 10^−186^).

### Reporting Summary

Further information on research design is available in the Nature Research Reporting Summary linked to this article.

## Online content

Any methods, additional references, Nature Research reporting summaries, source data, extended data, supplementary information, acknowledgements, peer review information, details of author contributions and competing interests, and statements of code and data availability are available at 10.1038/s41592-019-0658-6.

## Supplementary information

Supplementary Information Supplementary Figs. 1–13, Tables 1–9 and Notes 1–9.

Reporting Summary

Supplementary Table 4 Class-wise score for the nine invited teams, Macro F1 score per class for each of the invited teams in the competition.

Supplementary Table 5 Models and ablation study from the nine selected teams, Description of the different models used by the invited teams as well as an analysis of what factors contributed the most to the performance of the models.

## Data Availability

The dataset used for the HPA competition is available at: https://www.kaggle.com/c/human-protein-atlas-image-classification. The external dataset HPAv18 is publicly available on the HPA: https://v18.proteinatlas.org/. A script is provided for downloading the dataset is available at https://github.com/CellProfiling/HPA-competition.

## Code availability

Source code used to produce the figures has been released under permissive licenses at https://github.com/CellProfiling/HPA-competition. A HPA classification competition model zoo is being built to offer downloads of the top models generated during the competition. The model zoo can be found at https://modelzoo.cellprofiling.org.

The source code for the ImJoy plugin HPA-UMAP can be found at https://github.com/imjoy-team/example-plugins.
