# Author Correction: Analysis of the Human Protein Atlas Image Classification competition

**DOI:** 10.1038/s41592-020-0937-2

**Published:** 2020-08-05

**Authors:** Wei Ouyang, Casper F. Winsnes, Martin Hjelmare, Anthony J. Cesnik, Lovisa Åkesson, Hao Xu, Devin P. Sullivan, Shubin Dai, Jun Lan, Park Jinmo, Shaikat M. Galib, Christof Henkel, Kevin Hwang, Dmytro Poplavskiy, Bojan Tunguz, Russel D. Wolfinger, Yinzheng Gu, Chuanpeng Li, Jinbin Xie, Dmitry Buslov, Sergei Fironov, Alexander Kiselev, Dmytro Panchenko, Xuan Cao, Runmin Wei, Yuanhao Wu, Xun Zhu, Kuan-Lun Tseng, Zhifeng Gao, Cheng Ju, Xiaohan Yi, Hongdong Zheng, Constantin Kappel, Emma Lundberg

**Affiliations:** 1 Science for Life Laboratory, School of Engineering Sciences in Chemistry, Biotechnology and Health, KTH–Royal Institute of Technology, Stockholm, Sweden; 2 Department of Genetics, Stanford University, Stanford, CA USA; 3 Chan Zuckerberg Biohub, San Francisco, CA USA; 4 Changsha, China; 5 Winning Health Technology Group Co., Ltd., Shanghai, China; 6 Seoul, Republic of South Korea; 7 Missouri University of Science and Technology, Rolla, MO USA; 8 Khumbu.ai, Munich, Germany; 9 Qualcomm, Inc., Cupertino, CA USA; 10 Brisbane, Queensland Australia; 11 H2O.ai, Greencastle, IN USA; 12 SAS Institute, Inc., Cary, NC USA; 13 Jilian Technology Group (Video++), Shanghai, China; 14 SAP, Moscow, Russian Federation; 15 BDO Unicon, Saint Petersburg, Russian Federation; 16 Ivanovo, Russian Federation; 17 Kharkiv National University of Radioelectronics, Kharkiv, Ukraine; 18 Santa Clara, CA USA; 19 UT MD Anderson Cancer Center, Houston, TX USA; 20 Shanghai, China; 21 University of Hawaii Cancer Center, Honolulu, HI USA; 22 Taipei, Taiwan; 23 Microsoft Research, Beijing, China; 24 University of California Berkeley, Berkeley, CA USA; 25 Beijing, China; 26 Peking University, Beijing, China; 27 Leica Microsystems, Mannheim, Germany

**Keywords:** Organelles, Machine learning, Image processing

Correction to: *Nature Methods* 10.1038/s41592-019-0658-6, published online 28 November 2019.

In the version of this article initially published, the institution name was missing from author Dmytro Panchenko’s affiliation. The full affiliation is Kharkiv National University of Radioelectronics, Kharkiv, Ukraine. The error has been corrected in the PDF and HTML versions of the article.

