# Supplementary material for: Analysis of the Human Protein Atlas Image Classification competition
Source: Nat Methods. 2019 Nov 28;16(12):1254–61. doi: 10.1038/s41592-019-0658-6 (PMC6976526; doi:10.1038/s41592-019-0658-6)
Supplement: Supplementary file 1 — Supplementary Figs. 1–13, Tables 1–9 and Notes 1–9. [file 41592_2019_658_MOESM1_ESM.pdf]

https://doi.org/10.1038/s41592-019-0658-6

Analysis of the Human Protein Atlas Image Classification competition

Wei Ouyang[1], Casper F. Winsnes[1], Martin Hjelmare[1], Anthony J. Cesnik[2,3], Lovisa Åkesson[1], Hao Xu[1], Devin P. Sullivan[1], Shubin Dai[4], Jun Lan[5], Park Jinmo[6], Shaikat M. Galib[7], Christof Henkel[8], Kevin Hwang[9], Dmytro Poplavskiy[10], Bojan Tunguz[11], Russel D. Wolfinger[12], Yinzheng Gu[13], Chuanpeng Li[13], Jinbin Xie[13], Dmitry Buslov[14], Sergei Fironov[15], Alexander Kiselev[16], Dmytro Panchenko[17], Xuan Cao[18], Runmin Wei[19], Yuanhao Wu[20], Xun Zhu[21], Kuan-Lun Tseng[22], Zhifeng Gao[23], Cheng Ju[24], Xiaohan Yi[25], Hongdong Zheng[26], Constantin Kappel[27] and Emma Lundberg[1,2,3]*

[1]Science for Life Laboratory, School of Engineering Sciences in Chemistry, Biotechnology and Health, KTH–Royal Institute of Technology, Stockholm, Sweden. [2]Department of Genetics, Stanford University, Stanford, CA, USA. [3]Chan Zuckerberg Biohub, San Francisco, CA, USA. [4]Changsha, China. [5]Winning Health Technology Group Co., Ltd., Shanghai, China. [6]Seoul, Republic of South Korea. [7]Missouri University of Science and Technology, Rolla, MO, USA. [8]Khumbu.ai, Munich, Germany. [9]Qualcomm, Inc., Cupertino, CA, USA. [10]Brisbane, Queensland, Australia. [11]H2O.ai, Greencastle, IN, USA. [12]SAS Institute, Inc., Cary, NC, USA. [13]Jilian Technology Group (Video++), Shanghai, China. [14]SAP, Moscow, Russian Federation. [15]BDO Unicon, Saint Petersburg, Russian Federation. [16]Ivanovo, Russian Federation. [17]Kharkiv National University of Radioelectronics, Kharkiv, Ukraine. [18]Santa Clara, CA, USA. [19]UT MD Anderson Cancer Center, Houston, TX, USA. [20]Shanghai, China. [21]University of Hawaii Cancer Center, Honolulu, HI, USA. [22]Taipei, Republic of China. [23]Microsoft Research, Beijing, China. [24]University of California Berkeley, Berkeley, CA, USA. [25]Beijing, China. [26]Peking University, Beijing, China. [27]Leica Microsystems, Mannheim, Germany. *e-mail: emma.lundberg@scilifelab.se

Supplementary Information for

Analysis of the Human Protein Atlas Image Classification Competition

Ouyang et. al, Nature Methods, 2019

Supplementary Figure 1: F1 score per cell type for top 10 teams **3**

Supplementary Figure 2: Confusion matrices for the winning model prediction **6**

Supplementary Figure 3: Model architecture for Team 1 (bestfitting) **6**

Supplementary Figure 4: Model architecture for Team 2 (WAIR) **7**

Supplementary Figure 5: Model architecture for Team 3 (pudae) **8**

Supplementary Figure 6: Model architecture for Team 4 (Wienerschnitzelgemeinschaft) **9**

Supplementary Figure 7: Model architecture for Team 5 (VPP) **10**

Supplementary Figure 8: Model architecture for Team 8 (One more layer of stacking) **11**

Supplementary Figure 9: Model architecture for Team 10 (Conv is all you need) **12**

Supplementary Figure 10: Model architecture for Team 16 (NTU_MiRA) **13**

Supplementary Figure 11: Model architecture for Team 39 (Random Walks) **14**

Supplementary Figure 12: Overview of the models used to generate feature visualization and class activation maps **15**

Supplementary Figure 13: Assessment of biological relevance of the winning model with class activation maps **16**

Supplementary Table 1: List of merged classes for the challenge **17**

Supplementary Table 2: Image counts for each localization pattern **18**

Supplementary Table 3: Image counts for each cell line **19**

Supplementary Table 4: Class-wise score for the 9 invited teams (See attached .xlsx file) **19**

Supplementary Table 5: Models and ablation study from the 9 selected teams (See attached .xlsx file) **19**

Supplementary Table 6: Class-wise performance boost by adding HPAv18 for training **20**

Supplementary Table 7: Ensembling using models from the top 3 teams **21**

Supplementary Table 8: 5-fold cross-validation for the winning model of Team 1 **21**

Supplementary Table 9: Statistics for the violin plot elements in Fig. 2c and Fig. 2d **23**

Supplementary Notes: Team method descriptions **24**

1. Team 1 (Bestfitting) model summary 24

    Method Overview 24

The metric learning model 24
Ablation Study 24
Conclusions 25
2. Team 2 (WAIR) model summary 25
Method Overview 25
Ablation Study 26
Conclusions 26
3. Team 3 (Pudae) model summary 27
Method Overview 27
Ablation Study 27
Conclusions 28
4. Team 4 (Wienerschnitzelgemeinschaft) model summary 29
Method Overview 29
Ablation study 30
Conclusions 30
5. Team 5 (VPP) model summary 32
Method Overview 32
Ablation Study 32
Conclusions 33
6. Team 8 (One More Layer of Stacking) model summary 34
Method Overview 34
Ablation study 35
Conclusions 35
7. Team 10 (Conv is all you need) model summary 36
Method Overview 36
Ablation study 36
Conclusions 36
8. Team 16 (NTU_MiRA) model summary 38
Method Overview 38
Ablation Study 38
Conclusions 39
9. Team 39 (Random Walk) model summary 40
Method Overview 40
Ablation Study 40
Conclusions 40
References 41

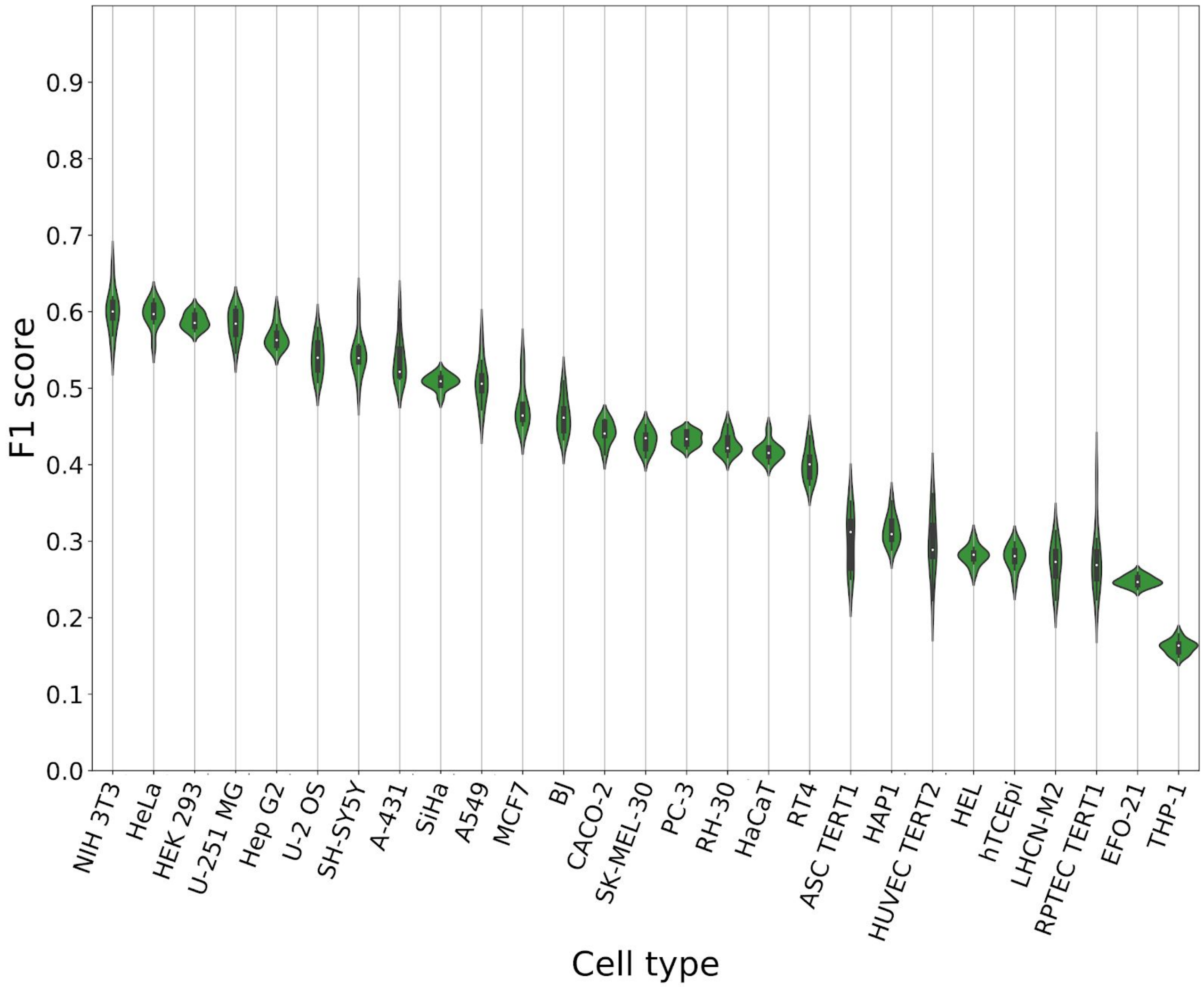

Supplementary Figure 1: F1 score per cell type for top 10 teams

Violin plot of the score distribution per cell type for the top 10 teams, ordered by decreasing median F1-score. n=10 teams for each violin. The minimum, mean, percentile (P), and maximum values are: NIH 3T3 (mean: 0.60, min: 0.55, 25th P: 0.59, 50th P: 0.60, 75th P: 0.61, max: 0.65), HeLa (mean: 0.60, min: 0.56, 25th P: 0.59, 50th P: 0.60, 75th P: 0.61, max: 0.62), HEK 293 (mean: 0.59, min: 0.57, 25th P: 0.58, 50th P: 0.59, 75th P: 0.60, max: 0.60), U-251 MG (mean: 0.58, min: 0.55, 25th P: 0.57, 50th P: 0.58, 75th P: 0.60, max: 0.61), Hep G2 (mean: 0.57, min: 0.55, 25th P: 0.56, 50th P: 0.56, 75th P: 0.57, max: 0.60), U-2 OS (mean: 0.54, min: 0.51, 25th P: 0.52, 50th P: 0.54, 75th P: 0.56, max: 0.58), SH-SY5Y (mean: 0.54, min: 0.50, 25th P: 0.53, 50th P: 0.54, 75th P: 0.55, max: 0.61), A-431 (mean: 0.54, min: 0.51, 25th P: 0.52, 50th P: 0.52, 75th P: 0.55, max: 0.60), SiHa (mean: 0.51, min: 0.49, 25th P: 0.50, 50th P: 0.51, 75th P: 0.51, max: 0.52), A549 (mean: 0.51, min: 0.46, 25th P: 0.50, 50th P: 0.51, 75th P: 0.52, max: 0.57), MCF7 (mean: 0.48, min: 0.45, 25th P: 0.46, 50th P: 0.46, 75th P: 0.48, max: 0.54), BJ (mean: 0.46, min: 0.43, 25th P: 0.44, 50th P: 0.46, 75th P: 0.47, max: 0.51), CACO-2 (mean: 0.44, min: 0.41, 25th P: 0.44, 50th P: 0.44, 75th P: 0.46, max: 0.46), SK-MEL-30 (mean: 0.43, min: 0.41, 25th P: 0.42, 50th P: 0.43, 75th P: 0.44, max: 0.45), PC-3 (mean: 0.43, min: 0.42, 25th P: 0.43, 50th P: 0.43, 75th P: 0.44, max: 0.44), RH-30 (mean: 0.43, min: 0.41, 25th P: 0.42, 50th P: 0.42, 75th P: 0.44, max: 0.45), HaCaT (mean: 0.42, min: 0.40, 25th P: 0.41, 50th P: 0.42, 75th P: 0.42, max: 0.45), RT4 (mean: 0.40, min: 0.37, 25th P: 0.38, 50th P: 0.40, 75th P: 0.41, max: 0.44), ASC TERT1 (mean: 0.30, min: 0.25,

25th P: 0.26, 50th P: 0.31, 75th P: 0.33, max: 0.35), HAP1 (mean: 0.31, min: 0.29, 25th P: 0.30, 50th P: 0.31, 75th P: 0.33, max: 0.35), HUVEC TERT2 (mean: 0.30, min: 0.22, 25th P: 0.28, 50th P: 0.29, 75th P: 0.32, max: 0.36), HEL (mean: 0.28, min: 0.26, 25th P: 0.28, 50th P: 0.28, 75th P: 0.28, max: 0.31), hTCEpi (mean: 0.28, min: 0.24, 25th P: 0.27, 50th P: 0.28, 75th P: 0.29, max: 0.30), RPTEC TERT1 (mean: 0.28, min: 0.22, 25th P: 0.25, 50th P: 0.27, 75th P: 0.29, max: 0.39), LHCN-M2 (mean: 0.27, min: 0.22, 25th P: 0.25, 50th P: 0.27, 75th P: 0.29, max: 0.31), EFO-21 (mean: 0.25, min: 0.24, 25th P: 0.24, 50th P: 0.25, 75th P: 0.25, max: 0.26), THP-1 (mean: 0.16, min: 0.15, 25th P: 0.16, 50th P: 0.16, 75th P: 0.17, max: 0.18) .

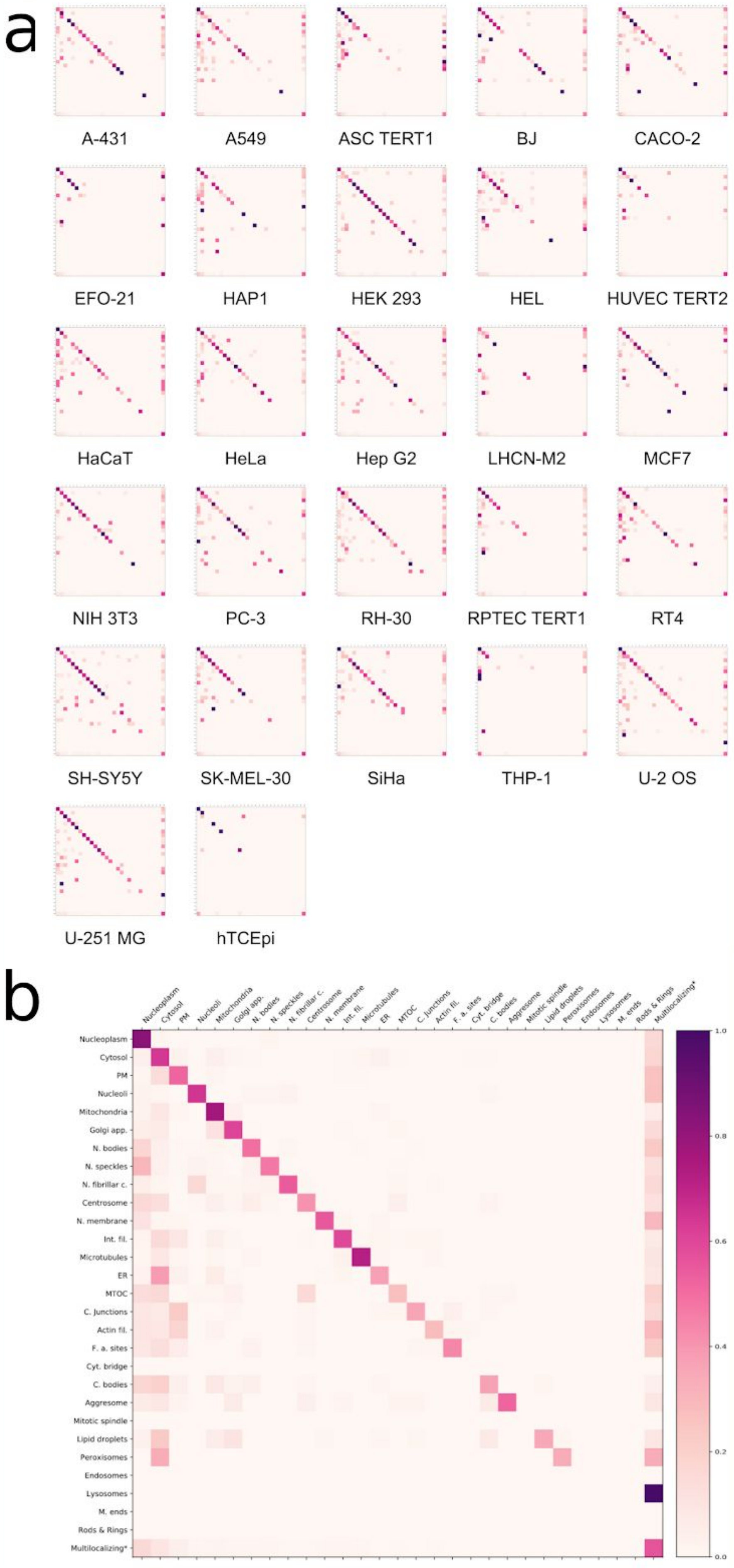
a
A-431
A549
ASC TERT1
BJ
CACO-2
EFO-21
HAP1
HEK 293
HEL
HUVEC TERT2
HaCaT
HeLa
Hep G2
LHCN-M2
MCF7
NIH 3T3
PC-3
RH-30
RPTEC TERT1
RT4
SH-SY5Y
SK-MEL-30
SiHa
THP-1
U-2 OS
U-251 MG
hTCEpi
b
Nucleoplasm
Cytosol
PM
Nucleoli
Mitochondria
Golgi app.
N. bodies
N. speckles
N. fibrillar c.
Centrosome
N. membrane
Int. fil.
Microtubules
ER
MTOC
C. Junctions
Actin fil.
F. a. sites
Cyt. bridge
C. bodies
Aggresome
Mitotic spindle
Lipid droplets
Peroxisomes
Endosomes
Lysosomes
M. ends
Rods & Rings
Multilocalizing*
1.0
0.8
0.6
0.4
0.2
0.0

Supplementary Figure 2: Confusion matrices for the winning model prediction

Confusion matrices for all cell lines combined **(a)** and individually **(b)** and combined **(a)** are calculated for samples in the 'test_private' set based on the predictions of the winning model (Team 1). Ground truth annotations are displayed on the Y-axis and model predictions on the X-axis. Due to the multilabel nature of the dataset, a special class "Multilocalizing*" which represents all possible combinations of multiple classes, is added. Notice that a high value in the multilocalizing column does not necessarily mean that the model is confused about that class but rather that the model associates that kind of images with multilabel annotations. Labels, with the exception of "Multilocalizing*", are sorted in descending order of rarity. Both "Cytosol" and "Nucleoplasm" show clear overannotation, with the model especially confusing "ER" and "Peroxisomes" for "Cytosol". The order of the labels and axes in each sub panel in **(a)** are the same as **(b)**.

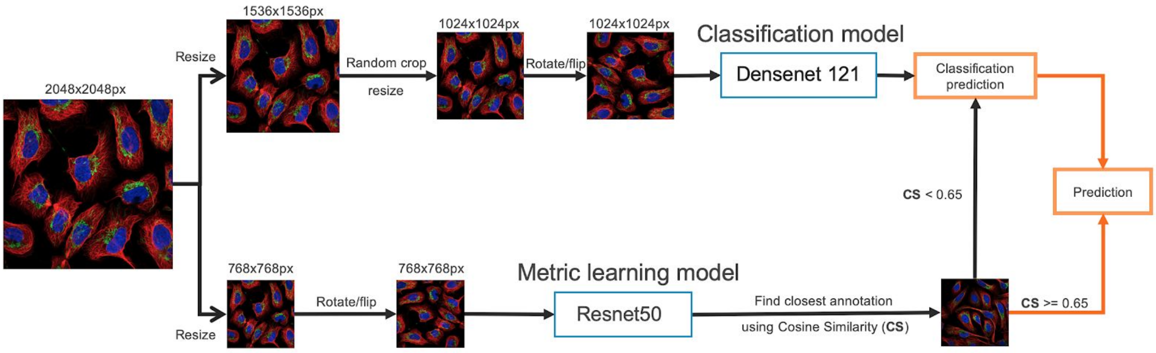

Supplementary Figure 3: Model architecture for Team 1 (bestfitting)

The model from team 1 consists of two parts: a classification model (Densenet 121, top) to perform classification; A separate metric learning model (Resnet50, bottom) is used to find the closest (measured in Cosine Similarity, labeled as CS) image from the HPAv18 dataset. For every test image, if the Cosine Similarity value is greater than 0.65, the predicted labels from the classification model will be replaced with the labels of the closest image in HPAv18.

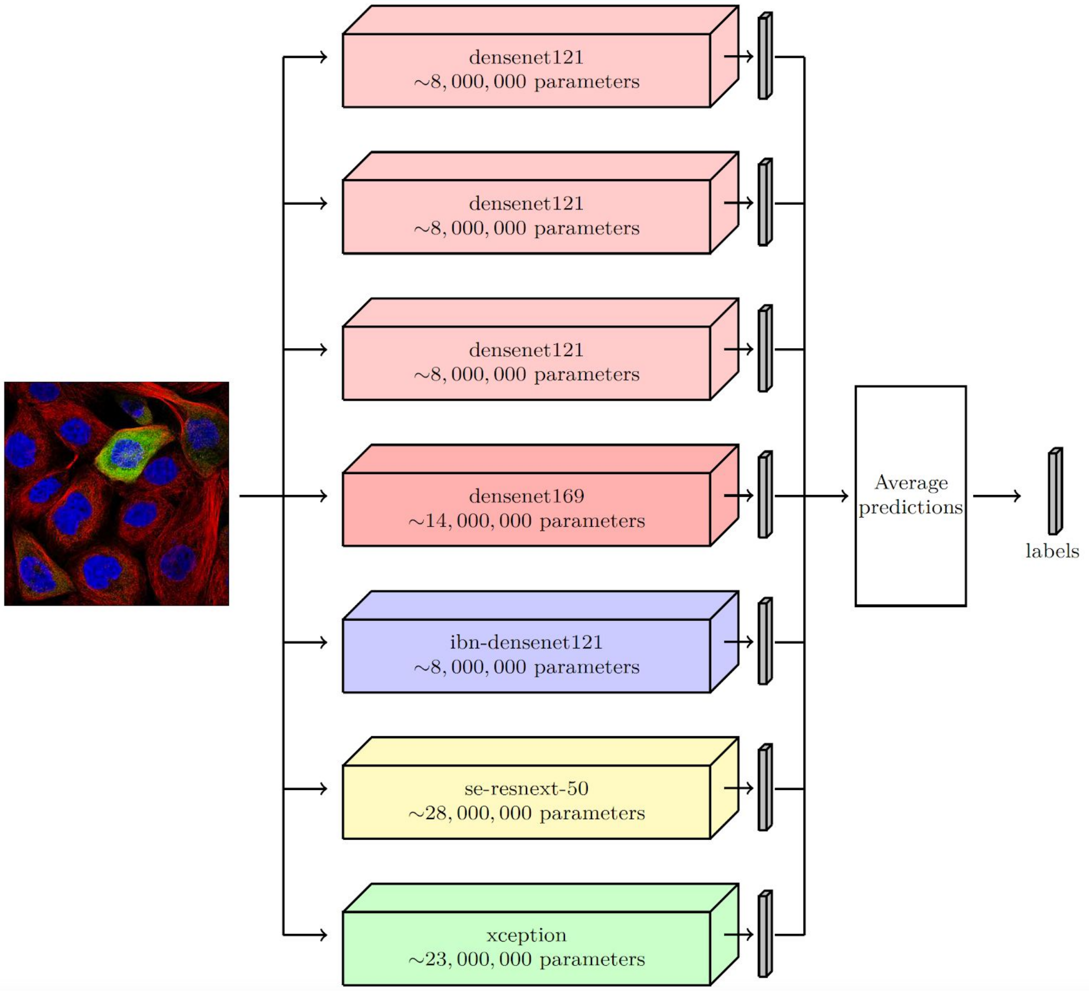

Supplementary Figure 4: Model architecture for Team 2 (WAIR)

Team 2 used an ensemble of models. Predictions from each of the neural network models are averaged into a single prediction.

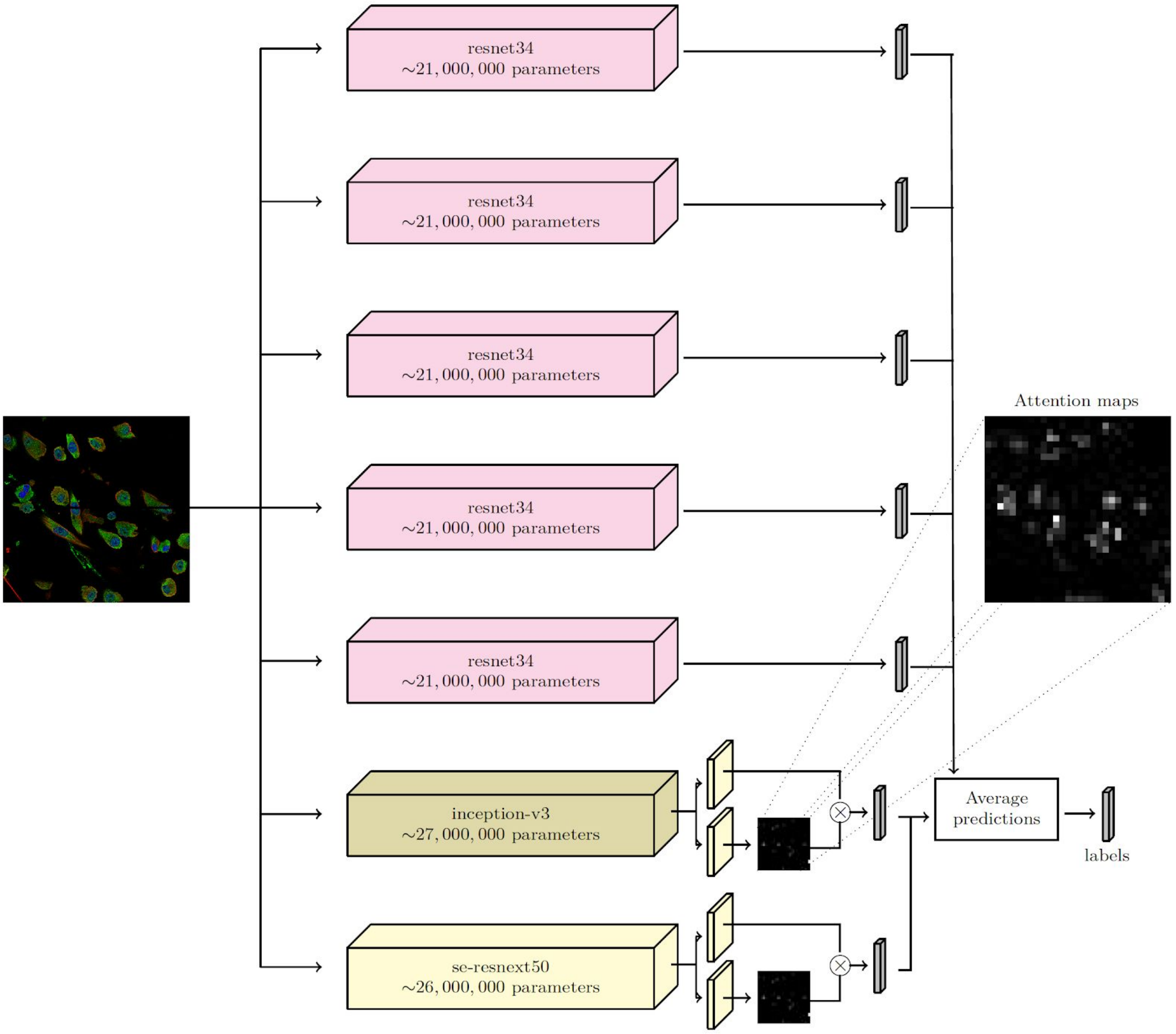

Supplementary Figure 5: Model architecture for Team 3 (pudae)

Team 3 used an ensemble of models. Predictions from the attention gated networks are combined with predictions from regular neural networks by averaging the two approaches' predictions.

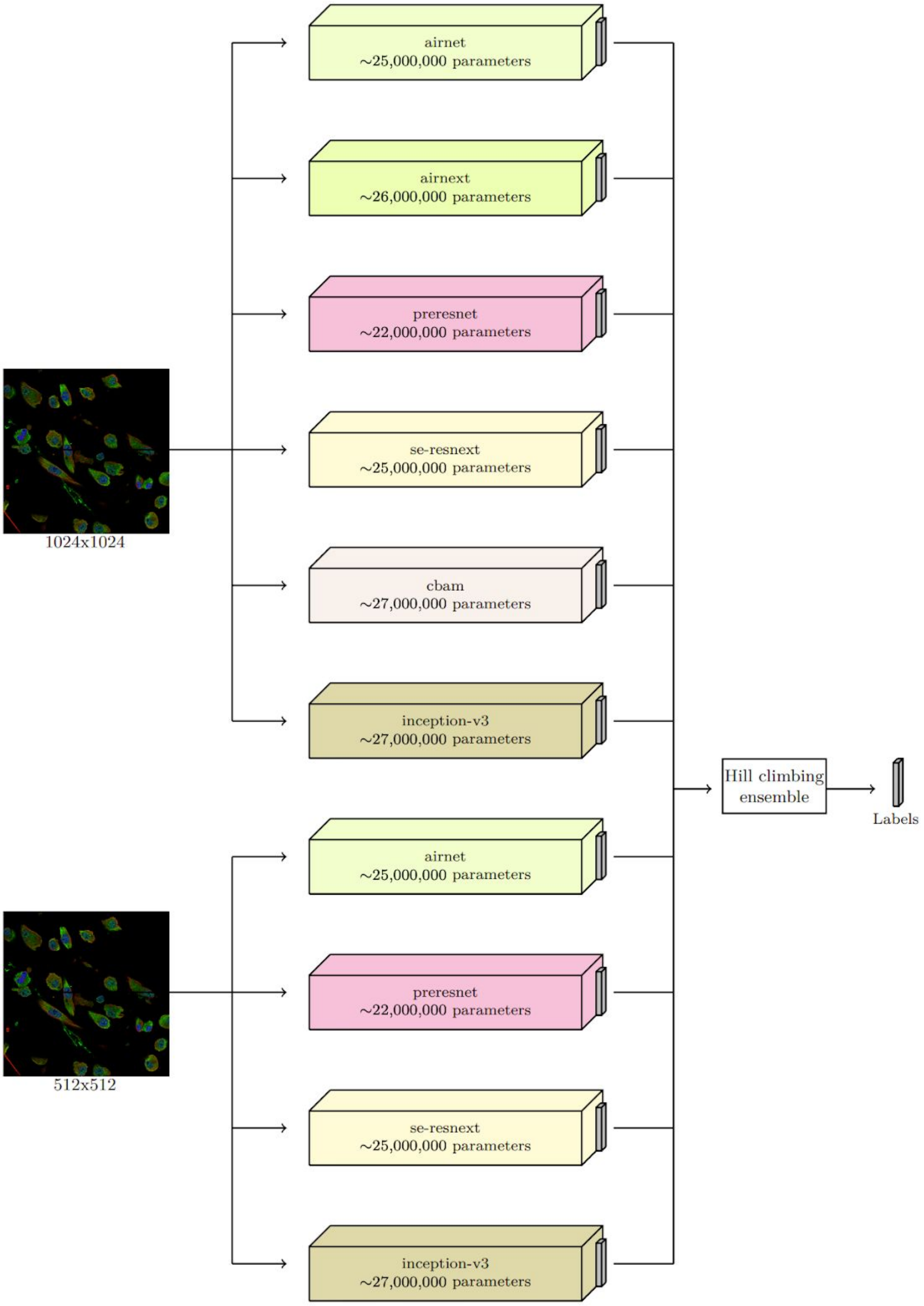

Supplementary Figure 6: Model architecture for Team 4 (Wienerschnitzelgemeinschaft)

Team 4 used an ensemble of models. Predictions from each of the neural network models are combined using a hill climbing algorithm.

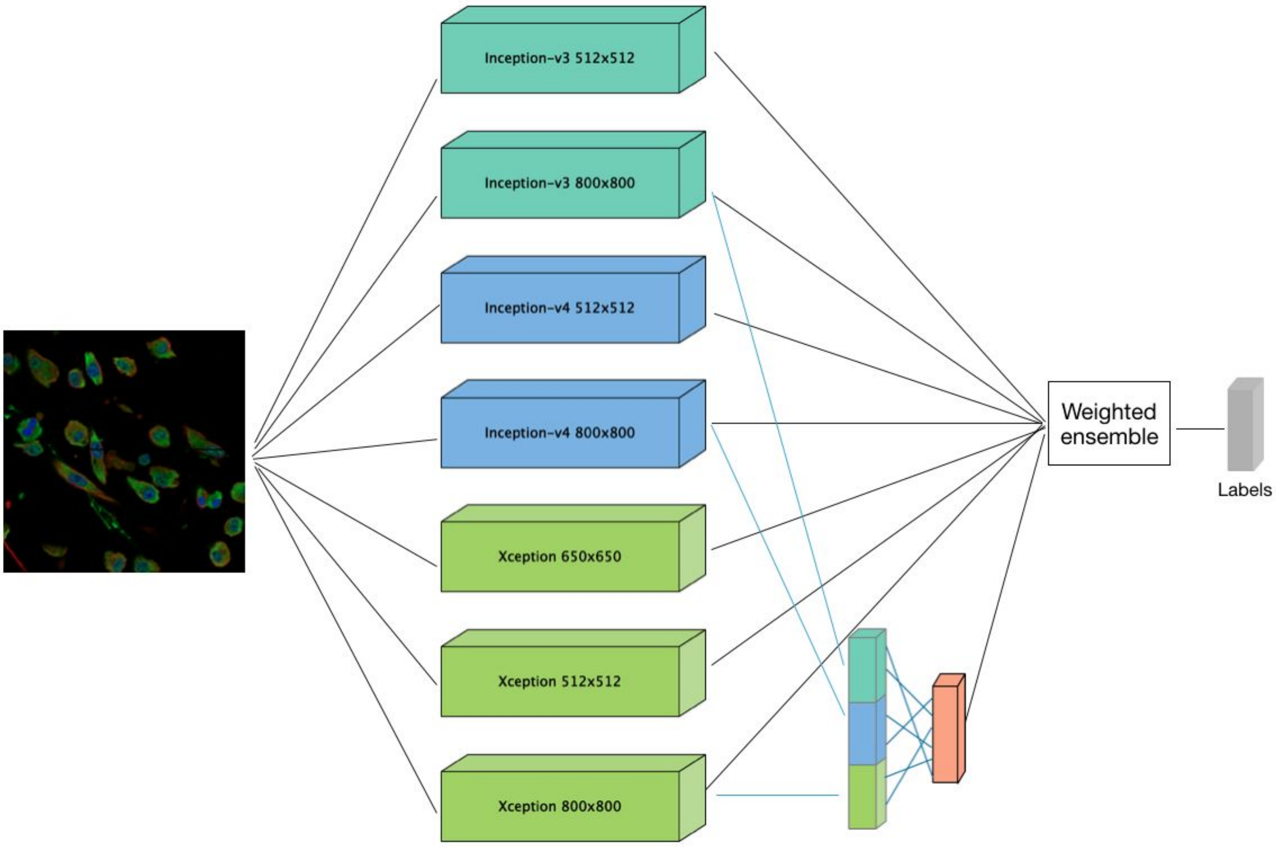

Supplementary Figure 7: Model architecture for Team 5 (VPP)

Team 5 used an ensemble of models. Predictions from each of the neural network models are combined using stacking and weighted ensembling.

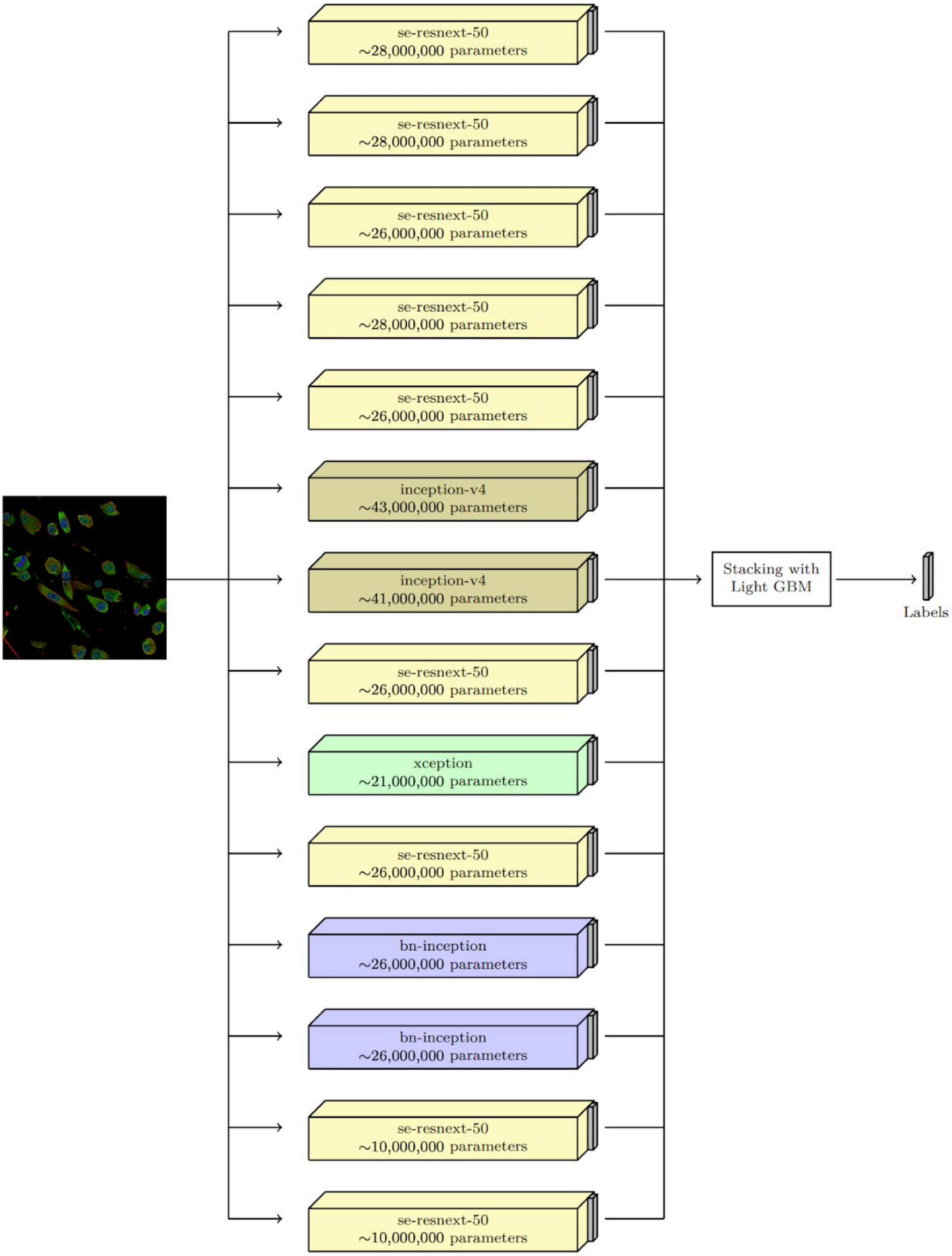

Supplementary Figure 8: Model architecture for Team 8 (One more layer of stacking)

Team 8 used an ensemble of models. Multiple neural networks are combined using a per-class voting ensemble.

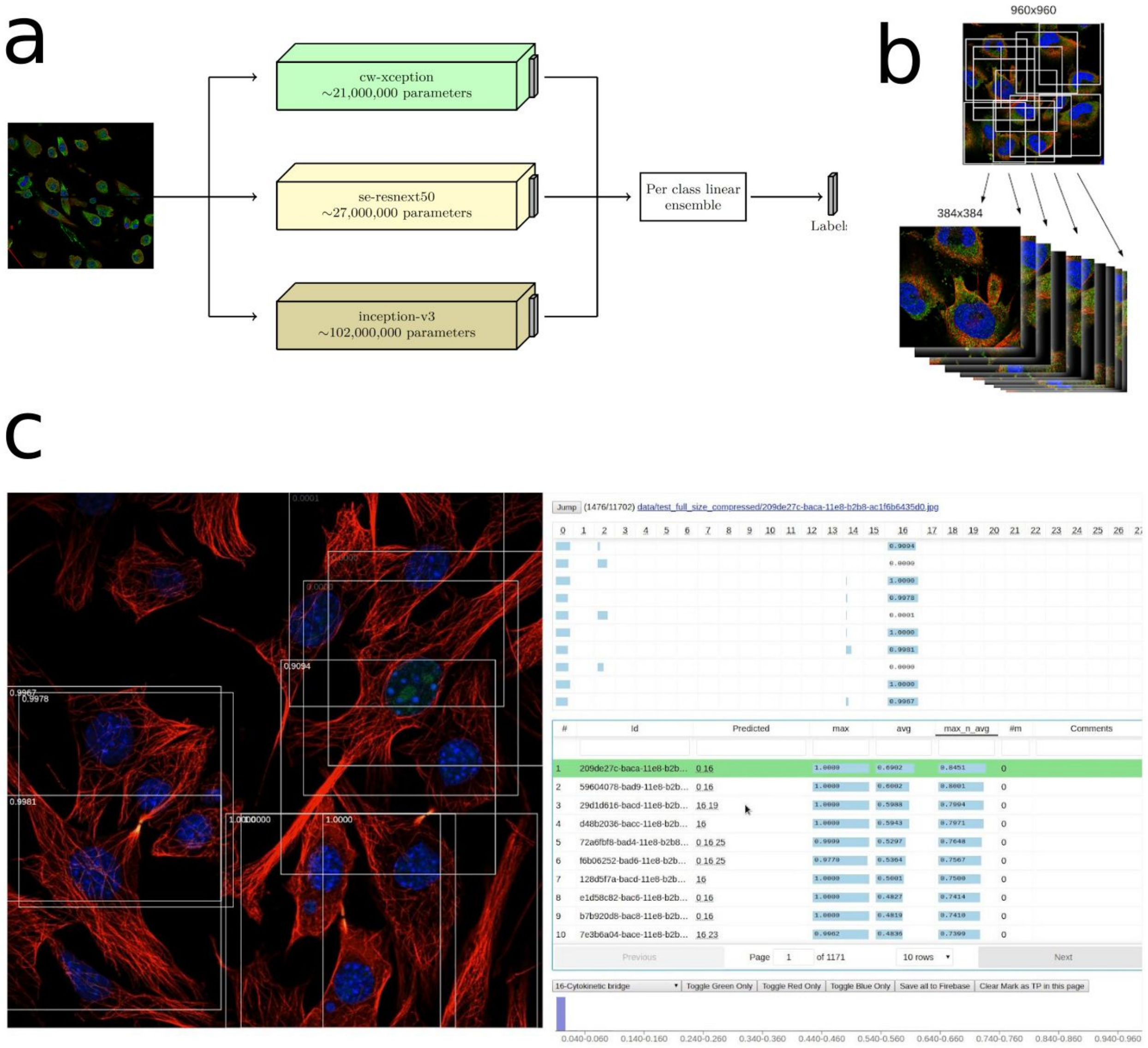

Supplementary Figure 9: Model architecture for Team 10 (Conv is all you need)

Team 10 used an ensemble of models. Three neural networks are combined using linear ensembling. (**a**); a target based cropping window technique was used (**b**) and (**c**) shows a screenshot of the GUI tool designed by the team.

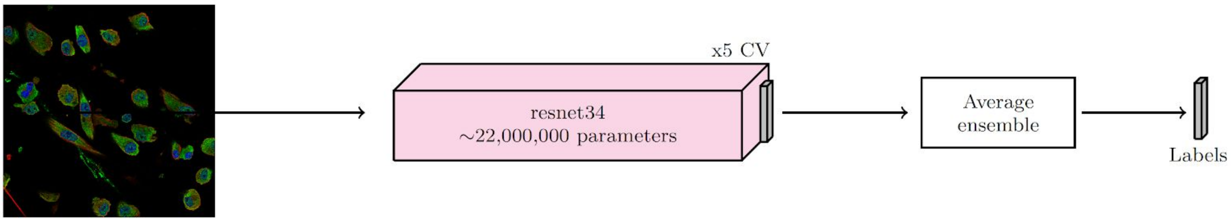

Supplementary Figure 10: Model architecture for Team 16 (NTU_MiRA)

Team 16 ensembled 5 cross-validated fold outputs by averaging their values.

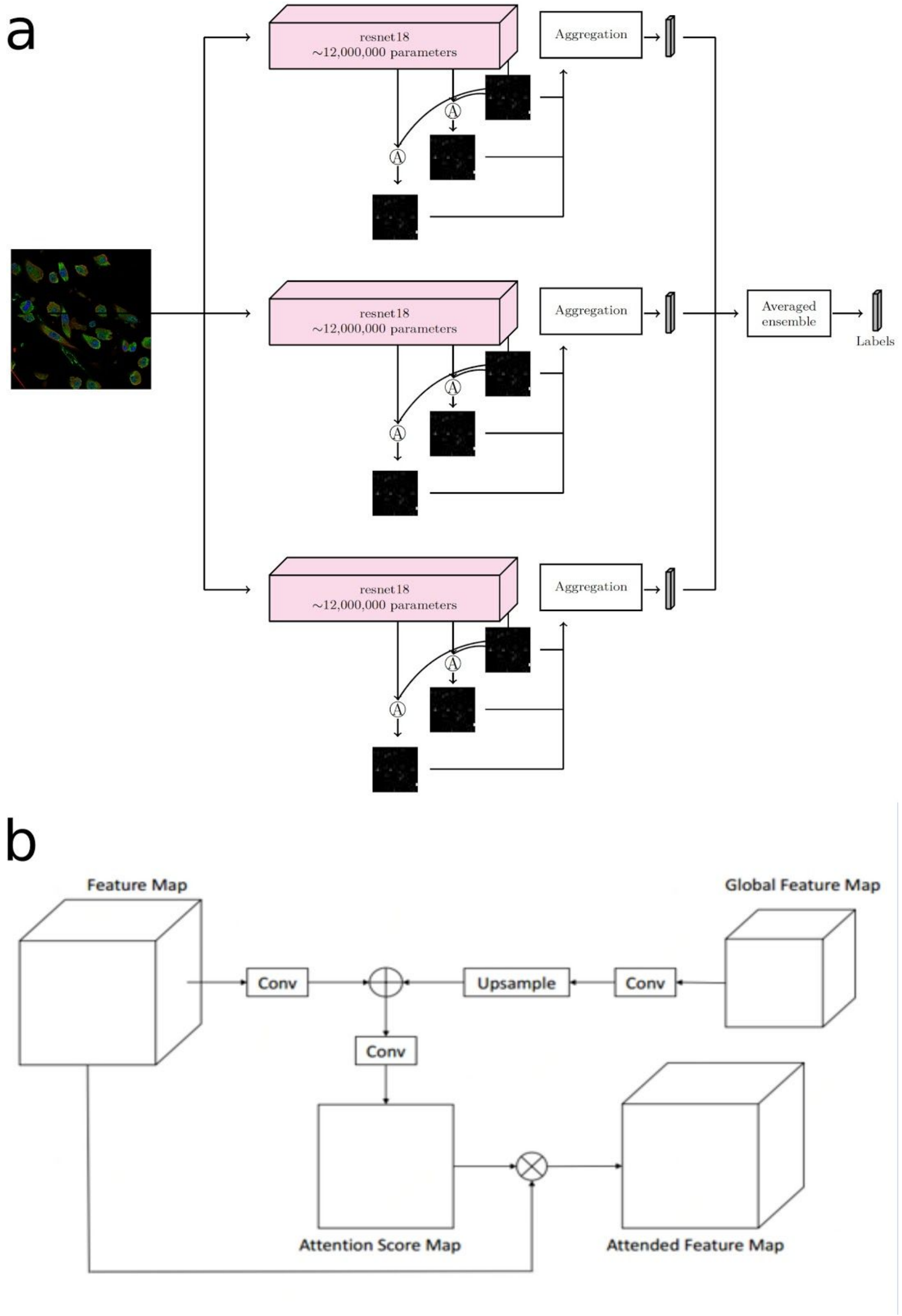

Supplementary Figure 11: Model architecture for Team 39 (Random Walks)

(a) Three attention gated neural networks, using ResNet18 as the backbone architecture, are ensembled through averaging each model's aggregated output. Each circled A in the figure represents an attention gate; (b) Attention mechanism of Random Walk's model.

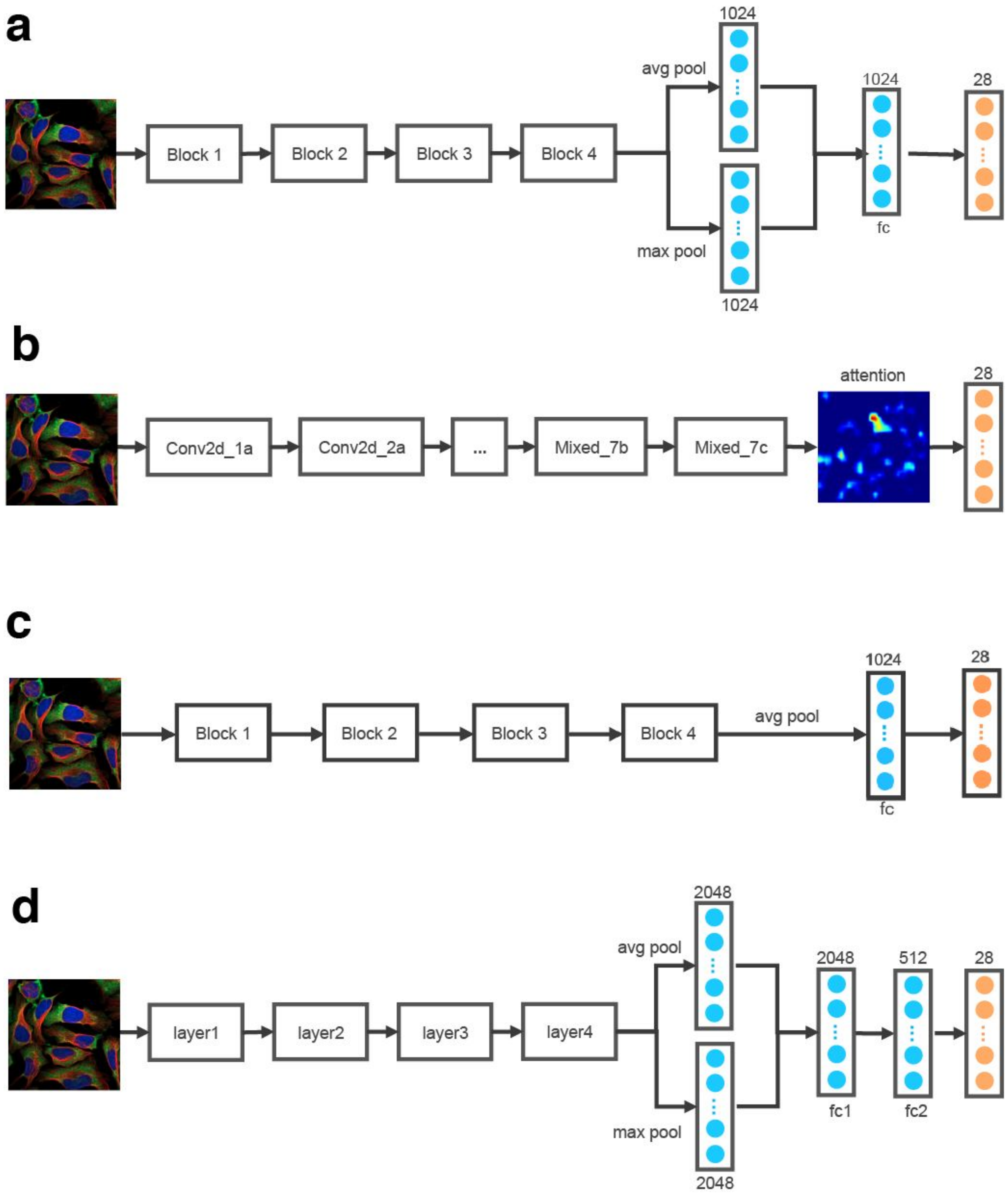

Supplementary Figure 12: Overview of the models used to generate feature visualization and class activation maps

a) densenet121_1024 model from Team 1. **b)** inception_v3 model from Team 3. **c)** densenet121_standard_no_crop_model from Team 1. **d)** resnet50 metric learning model from Team 1.

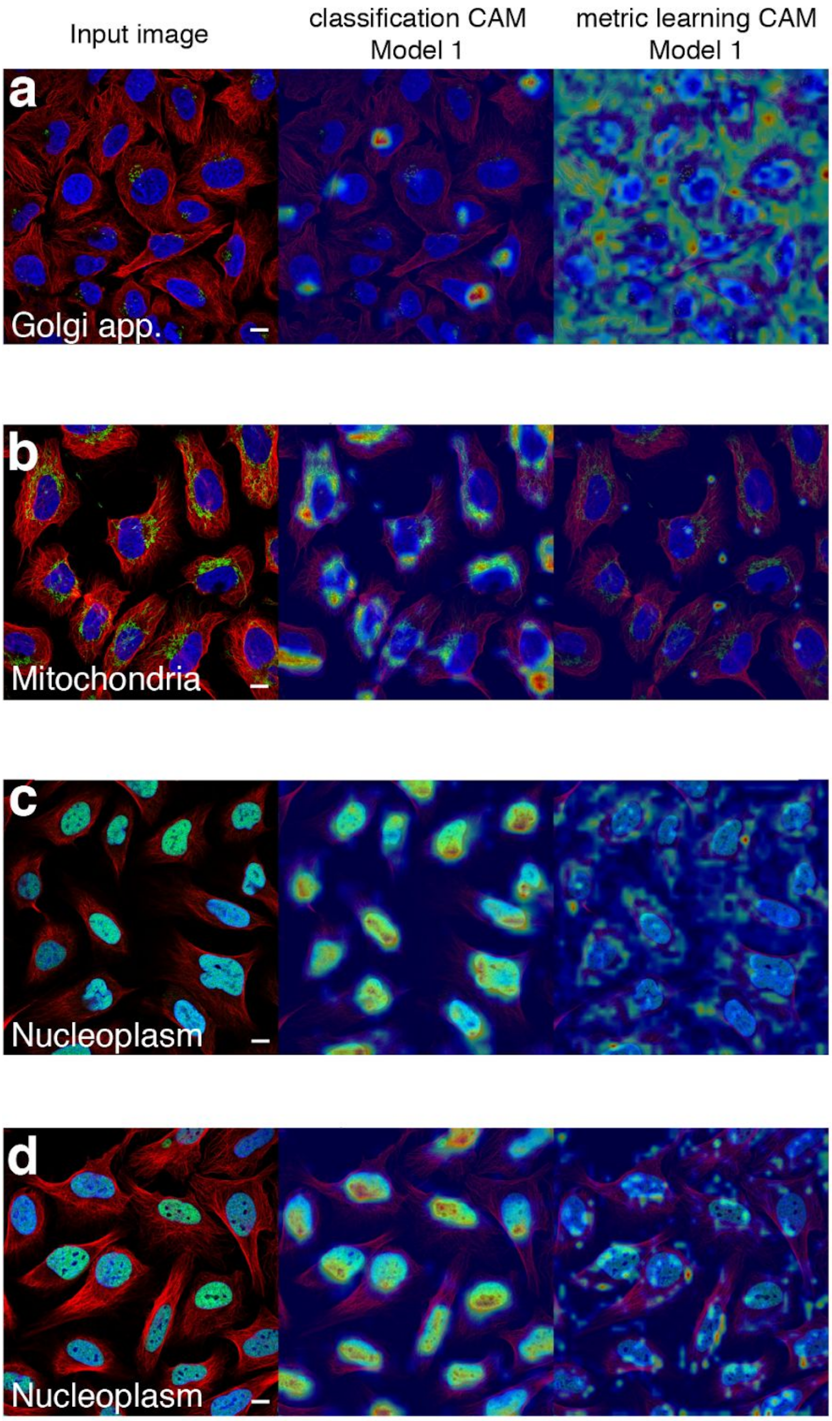

Supplementary Figure 13: Assessment of biological relevance of the winning model with class activation maps

Comparison of class activation maps (CAM) for the classification model and the metric learning model from Team 1. **a)** The protein Golgi reassembly stacking protein 1 localized to the Golgi apparatus. **b)** Citrate synthase localized to the mitochondria. **c)** PDS5 cohesin associated factor A localized to the Nucleoplasm. **d)** TATA-box binding protein associated factor 15 localized to the Nucleoplasm. To the left is the input image with the protein of interest shown in green, and the reference channels for microtubules in red and nucleus in blue. Scale bar 10 μm.

Class index	Kaggle Class	Merged HPA Class	Abbreviated Class Name
0	Nucleoplasm	Nucleus; Nucleoplasm	Nucleoplasm
1	Nuclear membrane	Nuclear membrane	N. membrane
2	Nucleoli	Nucleoli	Nucleoli
3	Nucleoli fibrillar center	Nucleoli fibrillar center	N. fibrillar c.
4	Nuclear speckles	Nuclear speckles	N. speckles
5	Nuclear bodies	Nuclear bodies	N. bodies
6	Endoplasmic reticulum	Endoplasmic reticulum	ER
7	Golgi apparatus	Golgi apparatus	Golgi app.
8	Peroxisomes	Peroxisomes	Peroxisomes
9	Endosomes	Endosomes	Endosomes
10	Lysosomes	Lysosomes	Lysosomes
11	Intermediate filaments	Intermediate filaments	Int. fil.
12	Actin filaments	Actin filaments	Actin fil.
13	Focal adhesion sites	Focal adhesion sites	F. a. sites
14	Microtubules	Microtubules	Microtubules
15	Microtubule ends	Microtubule ends	M. ends
16	Cytokinetic bridge	Cytokinetic bridge; Midbody; Midbody ring; Cleavage furrow	Cyt. bridge
17	Mitotic spindle	Mitotic spindle	Mitotic spindle
18	Microtubule organizing center	Microtubule organizing center; Centriolar satellite	MTOC
19	Centrosome	Centrosome	Centrosome
20	Lipid droplets	Lipid droplets	Lipid droplets
21	Plasma membrane	Plasma membrane	PM
22	Cell junctions	Cell junctions	C. Junctions
23	Mitochondria	Mitochondria	Mitochondria
24	Aggresome	Aggresome	Aggresome
25	Cytosol	Cytosol	Cytosol
26	Cytoplasmic bodies	Cytoplasmic bodies	C. bodies
27	Rods & Rings	Rods & Rings	Rods & Rings

Supplementary Table 1: List of merged classes for the challenge

Certain classes within the HPA were merged into a single class for the challenge. Listed here are all the classes, including index, used in the Kaggle challenge and what HPA classes that were merged to form the Kaggle classes. We also list the abbreviated class names that we used in plots in this paper.

Localization	HPAv18	Training	Test	Validation_public	Private_test
Nucleoplasm	31590	12885	4396	1251	3083
Cytosol	22596	8228	2724	810	1876
PM	6590	3777	1578	451	1117
Nucleoli	5186	3621	1058	287	764
Mitochondria	5794	2965	930	302	624
Golgi app.	4058	2822	1031	300	728
N. bodies	2316	2513	922	282	637
N. speckles	2708	1858	891	270	619
N. fibrillar c.	1484	1561	770	250	519
Centrosome	1488	1482	810	250	553
N. membrane	1260	1254	708	183	523
Int. fil.	628	1093	621	168	450
Microtubules	1244	1066	697	184	495
ER	1988	1008	591	165	426
MTOC	494	902	503	140	359
C. Junctions	1004	802	426	99	325
Actin fil.	998	688	469	153	315
F. a. sites	564	537	296	92	204
Cyt. bridge	438	530	468	97	254
C. bodies	302	328	453	122	331
Aggresome	156	322	162	41	121
Mitotic spindle	60	210	314	84	208
Lipid droplets	158	172	145	40	105
Peroxisomes	134	53	14	5	9
Endosomes	94	45	17	8	9
Lysosomes	92	28	18	8	10
M. ends	92	21	7	5	2
Rods & Rings	42	11	13	10	3

Supplementary Table 2: Image counts for each localization pattern

Shown are the image counts in the HPAv18, training and test sets, ordered by class labels. Note that for some classes the public and private test sets do not sum up to the same number as the Test column due to having images discarded during the leak correction.

Cell Line	Training	Test
U-2 OS	2973	646
U-251 MG	2596	577
A-431	2254	505
RH-30	1872	559
HEK 293	1737	498
NIH 3T3	1695	518
SiHa	1674	562
PC-3	1662	451
HEL	1514	517
SH-SY5Y	1457	460
CACO-2	1415	472
HeLa	1389	509
A549	1371	569
Hep G2	1198	409
HaCaT	985	482
MCF7	923	416
BJ	902	420
RT4	823	463
SK-MEL-30	799	423
ASC TERT1	498	417
RPTEC TERT1	349	391
HAP1	328	358
HUVEC TERT2	195	345
EFO-21	192	248
LHCN-M2	108	232
hTCEpi	89	67
THP-1	74	188

Supplementary Table 3: Image counts for each cell line

Shown are the image counts in the training and test sets, ordered by cell line training count.

Supplementary Table 4: Class-wise score for the 9 invited teams
(See attached .xlsx file)

Macro-F1 score per class for each of the invited teams in the competition.

Supplementary Table 5: Models and ablation study from the 9 selected teams
(See attached .xlsx file)

Description of the different models used by the invited teams as well as an analysis of what factors contributed the most to the performance of the models.

Class name	Densenet121 trained without HPAv18 (macro F1=0.510)	Densenet121 trained with HPAv18 (macro F1=0.552)	F1 Score improvements
Nucleoplasm	0.76	0.777	0.017
Nuclear membrane	0.722	0.759	0.037
Nucleoli	0.718	0.718	0
Nucleoli fibrillar center	0.626	0.645	0.019
Nuclear speckles	0.597	0.648	0.051
Nuclear bodies	0.539	0.558	0.019
Endoplasmic reticulum	0.529	0.552	0.023
Golgi apparatus	0.674	0.707	0.033
Peroxisomes	0.363	0.526	0.163
Endosomes	0.444	0.777	**0.333**
Lysosomes	0.428	0.625	0.197
Intermediate filaments	0.682	0.699	0.017
Actin filaments	0.55	0.563	0.013
Focal adhesion sites	0.554	0.575	0.021
Microtubules	0.738	0.727	-0.011
Microtubule ends	0	0	0
Cytokinetic bridge	0.342	0.376	0.034
Mitotic spindle	0.323	0.373	0.05
Microtubule organizing center	0.352	0.425	0.073
Centrosome	0.491	0.5	0.009
Lipid droplets	0.44	0.485	0.045
Plasma membrane	0.557	0.548	-0.009
Cell junctions	0.56	0.569	0.009
Mitochondria	0.658	0.676	0.018
Aggresome	0.666	0.676	0.01
Cytosol	0.542	0.548	0.006
Cytoplasmic bodies	0.412	0.437	0.025
Rods & Rings	0	0	0

Supplementary Table 6: Class-wise performance boost by adding HPAv18 for training

Comparing class-wise performance with the same Densenet-121 model trained with HPA v18 dataset or without. The model is from Team 1(bestfitting), also shown in Supplementary Table 5b, experiment 1, 3.

Model	Description	Private LB	Public LB
1	single model from team 1	0.565	0.627
2	ensemble of 7 models from team 2	0.571	0.641
3	ensemble of 3 models from team 3	0.57	0.614
ensemble	1:1:1 vote of model 1, 2 and 3	**0.575**	**0.654**

Supplementary Table 7: Ensembling using models from the top 3 teams

Using models from the top three teams results in an even better model.

Fold	Epoch	Validation score	Private LB	Public LB
1/5	35	0.5948	0.540	0.600
2/5	41	0.6120	0.549	0.604
3/5	41	0.6066	0.550	0.617
4/5	43	0.6073	0.542	0.620
5/5	41	0.5882	0.543	0.610

Supplementary Table 8: 5-fold cross-validation for the winning model of Team 1

The 5-fold cross validation experiments are performed from the winning model of team 1 (without the metric learning part), scores are shown for each fold, as shown in the table validation score (labeled as `Validation score`) varies from 0.5882 to 0.6120.

Violin plot statistics for Fig. 2c						
**Element name**	**Mean**	**Minimum**	**25% Percentile**	**50% Percentile**	**75% Percentile**	**Maximum**
Nucleoplasm	0.79	0.76	0.78	0.79	0.79	0.80
Cytosol	0.59	0.58	0.58	0.60	0.60	0.60
Plasma membrane	0.59	0.58	0.58	0.59	0.60	0.62
Nucleoli	0.73	0.69	0.72	0.73	0.74	0.76
Mitochondria	0.67	0.65	0.65	0.67	0.68	0.71
Golgi apparatus	0.69	0.67	0.68	0.69	0.70	0.72
Nuclear bodies	0.56	0.54	0.55	0.56	0.58	0.59
Nuclear speckles	0.64	0.60	0.62	0.64	0.66	0.67
Nucleoli fibrillar center	0.66	0.63	0.65	0.66	0.68	0.70
Endoplasmic reticulum	0.54	0.44	0.50	0.54	0.56	0.65
Centrosome	0.50	0.48	0.49	0.50	0.51	0.53
Nuclear membrane	0.74	0.73	0.73	0.74	0.76	0.76
Microtubules	0.76	0.72	0.74	0.75	0.77	0.78
Cell junctions	0.57	0.54	0.56	0.56	0.57	0.63
Intermediate filaments	0.70	0.67	0.68	0.69	0.71	0.75
Actin filaments	0.58	0.55	0.56	0.58	0.60	0.62
Microtubule organizing center	0.42	0.39	0.40	0.41	0.44	0.47
Focal adhesion sites	0.59	0.56	0.58	0.59	0.60	0.62
Cytokinetic bridge	0.39	0.36	0.37	0.39	0.40	0.44
Cytoplasmic bodies	0.41	0.33	0.38	0.42	0.43	0.44
Aggresome	0.64	0.60	0.64	0.64	0.65	0.69
Lipid droplets	0.50	0.44	0.47	0.49	0.51	0.57
Mitotic spindle	0.40	0.35	0.39	0.39	0.41	0.46
Peroxisomes	0.61	0.48	0.56	0.62	0.67	0.71
Endosomes	0.76	0.63	0.73	0.77	0.82	0.84
Lysosomes	0.78	0.71	0.75	0.76	0.82	0.86

Rods & Rings	0.04	0.00	0.00	0.00	0.00	0.40
Microtubule ends	0.05	0.00	0.00	0.00	0.00	0.50
**Violin plot statistics for Fig. 2d**						
**Element name**	**Mean**	**Minimum**	**25% Percentile**	**50% Percentile**	**75% Percentile**	**Maximum**
1-10, all	0.57	0.56	0.56	0.57	0.57	0.59
1-10, single	0.62	0.59	0.61	0.62	0.64	0.66
1-10, multi	0.55	0.54	0.54	0.55	0.55	0.58
11-100, all	0.54	0.52	0.53	0.53	0.54	0.56
11-100, single	0.58	0.54	0.57	0.58	0.60	0.62
11-100, multi	0.51	0.48	0.50	0.51	0.52	0.55
101-500, all	0.48	0.40	0.46	0.48	0.50	0.52
101-500, single	0.54	0.49	0.52	0.55	0.56	0.61
101-500, multi	0.46	0.38	0.45	0.46	0.48	0.52
501-2,137, all	0.31	0.00	0.21	0.37	0.43	0.46
501-2,137, single	0.37	0.00	0.27	0.45	0.50	0.56
501-2,137, multi	0.30	0.00	0.19	0.34	0.41	0.45

Supplementary Table 9: Statistics for the violin plot elements in Fig. 2c and Fig. 2d

The mean, minimum, 25%, 50%, 75% percentile, and maximum for the violin plot elements in Fig. 2c and 2d.

Supplementary Notes: Team method descriptions

1. Team 1 (Bestfitting) model summary

Method Overview

A CNN multi-label classification model was used to predict labels of each sample and a metric learning model to find the nearest sample for reference.

The CNN classification model

Multi-Label Stratification was used to split competition data and the HPAv18 dataset into a training set and a validation set. The performance of the model was estimated using focal loss over the validation set. A Densenet121 acts as the backbone of the model[3]. The *GlobalMaxPool* and *GlobalAvgPool* layers of the final CNN feature map were concatenated before being fed to two fully connected layers to calculate the probability of each class.

Image augmentation was performed by flipping the image, rotating it 90 degrees, and randomly cropping out 1024 $\times$ 1024 pixel (px) patches. To improve the model's predictive power, multiple random crops were taken, and the maximum probability among them was calculated when predicting on the test set.

A combined loss function of focal loss, Lovasz loss, and hard example log loss was used for training the model. It was optimized using an *Adam* optimizer with a step learning rate of [30, 15, 7.5, 3, 1] $\times$ 1e-5 for [25, 5, 5, 5, 5] epochs respectively[7]. The output was thresholded using the ratio of labels in the training set.

Using this method, the CNN reached a macro F1 of 0.565 on the private leaderboard when averaging predictions from 5 folds.

The metric learning model

A metric learning model was trained to, for a given sample, find the nearest sample available in the HPA v18 dataset. The approach was inspired by facial recognition technology, treating an antibody id as a "face" to be recognized, with *ArcFaceLoss* as the loss function[1,2]. A resNet50 architecture was used as backbone. One fourth of the samples from each antibody was used as a validation set and the rest were used as a training set. The images were resized to 768 $\times$ 768 px and augmented by flipping, rotating 90 degrees before being fed into the network. *Adam* was used as the optimizer with a learning rate of 1e-4 for 50 epochs when training.

The trained model calculates the similarities between any two given samples. When samples are highly similar (> 0.55), it is likely that the labels are similar to each other (> 0.91).

This information was used to enhance predictions made by the CNN model on images in the test set. If similar samples (similarity score > 0.65) could be found in the HPAv18 dataset, the prediction was replaced by the labels of the most similar sample. This step increased the macro F1 score on the private leaderboard to 0.593.

Ablation Study

This ablation study aims to understand how the data, validation strategies, loss functions, and network structures affected the model capabilities. The experiment numbers in this section refers to the indices in **Supplementary Table 5b**.

Experiment #2-5, #23-25, and #27-29 focused on data usage and augmentation options, and experiments #5-9 on the influences of different loss functions. Since this competition was focused on a multi-label classification task with extremely imbalanced labels, experiment #10 checked the gain of using MultilabelStratifiedKFold. Network structures were also investigated by comparing the heads of networks in experiments #11, #21, #25 with the dedicated head baseline of #1, #18, #22 accordingly. We also studied the different backbone options in #12 to #17, #18 and #22.

According to experiment #2, the addition of the yellow ER-channel input led to 0.013 gain on Private LB. The HPAv18 external dataset brought 0.031-0.042 improvement on Private LB. Experiment #4 confirmed that a good data augmentation strategy was important, as the improvement of 0.019 was significant; this strategy simulated the scale variance of images and different views in a batch of samples. Experiments #5-9 proved that the different loss functions were all important for the success of the final model. In particular, the Lovasz function, which is mainly used in image segmentation tasks, was helpful in this multi-label context as well. The Lovasz loss can balance the precision and recall, which helped to improve the F1 score, and since the F1 function is not differentiable, Lovasz loss is a good option.

Experiments #11, #21, and #25 show that modifications to the standard structure to densenet, resent, and inception v3 were important to this problem. From experiments #12-18, and #22, we can conclude that densenet121 is superior to other mainstream backbones. Densenet121 is strong enough to learn patterns from this dataset, and we do not need complex models such as Inception-resnet-v2 and Inception v4 to understand the patterns in the images. By comparing experiments #1 and #18, we can find that after finding a strong enough model, the score improvements from increasing the image size were very small; when the images increased from 512 × 512 px to 1024×1024 px, the score increased 0.0002.

Conclusions

Overall, the metric learning helped to improve the score in the competition. As for improving a CNN classification model, some factors seem more important than others, such as using the external HPA v18 dataset. Further increasing the number of available images by using random cropping also helped improve the models, mostly by reducing the risk of overfitting to the training set. We can also see how the addition of the yellow channel to the input helps the model find useful patterns. Choosing the right loss function is also important. A combination of several established loss functions let the models converge rapidly towards better performance, as compared to a single loss function. In particular, focal loss was helpful in estimating the model's capabilities so that training could be stopped at a reasonable epoch.

The network architecture plays an important role in the performance of the model. Modifying the structures of DenseNet and ResNet were important factors in handling the multiple labels in this classification task. It also seems that using too deep and complex networks reduced the performance, making the choice of the correct architectural backbone crucial. We also noticed that with a better model, the size of the input images becomes less important.

2. Team 2 (WAIR) model summary

Method Overview

An ensemble of CNN models using multiple different architectures was trained and evaluated on both the Kaggle competition data and the external HPA v18 dataset. Public tools used include PyTorch, Opencv, and scikit-learn.

Seven end-to-end models for handling the multi-label classification problem were created. A total of 5 different architectures were used across the seven models: 3 instances of densenet121, densenet169, ibn-densenet121, se-resnext50, and Xception[3,4,5,6,]. ImageNet pre-trained models were used as backbone models. The final fully connected layer for each model was replaced by a fully connected layer with 28 neurons, and the sigmoid activation function was connected to each of the 28 neurons to output the final predicted probability for each image class.

To train each model, Adamax was used as an optimizer, and a cycle learning rate with warm up was set up as a scheduler[7,8]. A customized weighted binary cross-entropy (BCE) loss was used as the final loss function. The cyclical learning rate technique was used and customized by implementing the following key factors: 1) Each model was trained for a total of 24 epochs. 2) The first 20 epochs were subjected to cosine annealing for learning rate decay, with learning rate oscillating from 0.001 to 0.00001 every 10 epochs. 3) The learning rate for epoch 21 and 22 was 1e-5 and the learning rate for epoch 23 and 24 was 1e-6. The threshold was determined by obtaining the maximum F1 score on local out-of-fold validation data and then scaled by a factor of 0.7. Finally, a prediction was made by averaging the predicted probabilities from each of the seven models.

There are three main points that were important for this solution. 1) First, a trick was used to add external data for training. The trick was based on finding similar images between the Kaggle and external data, and use the kaggle label as a reference to fetch the correct labels from the HPA website. Each image was first normalized then resized to 512 $\times$ 512 px which allowed for pixel histograms that could be compared to each other. 2) To handle the imbalanced classes, a carefully designed weighted BCE loss was used in conjunction with oversampling. 3) To obtain a good prediction threshold value, it was determined by what threshold obtained the maximum F1 score on a local out-of-fold validation. To make the model more robust, the threshold values were scaled by a factor of 0.7.

The training process took 20-30 hours for each model, and the full training took one week using four Nvidia GTX 1080Ti GPUs. The prediction process took around 10 hours for a single submission to Kaggle using one Nvidia GTX 1080Ti GPU.

Ablation Study

Several lessons were learned from comparing successive experiments. Experiment numbers in this section refers to the indices in **Supplementary Table 5d**. Increasing the image size from 256 $\times$ 256 to 512 $\times$ 512 px improved the performance, as seen when comparing experiments #1 and #2. Comparing experiments #2 and #3 showed that using 3 channels input (without yellow ER channel) can increase the performance of the model. Experiments #3 and #4 showed that searching thresholds increased the performance. Adding external data from HPAv18 significantly increased the performance, as shown in experiments #4 and #5. Finally, using weighted BCE loss and oversampling helped address the class imbalance problem and increased the performance between experiments #4 and #5.

Conclusions

Using external data (HPAv18) yielded a considerable increase in performance. Also, increasing the image size from 256 $\times$ 256 to 512 $\times$ 512 px was important for creating a more powerful model. Finally, the carefully designed weighted BCE loss and the application of oversampling were helpful in combating issues of class imbalance.

3. Team 3 (Pudae) model summary

Method Overview

An ensemble of regular models and attention gated CNN models were used. 7 different models were part of the ensemble: 5 resnet34 models, acting as the backbone of the model, 1 attention gated inception-v3, and 1 attention gated se-resnet50[5,11,12]. The final prediction is made by averaging the output from each of the models.

To address the class imbalance focal loss was used; it made other methods, such as under/oversampling, unnecessary[10]. Another difficulty caused by imbalanced data was evaluating the capability of a model since extremely rare classes made the macro F1 score unstable. To avoid this problem, the capability was estimated using the F1 score of the most frequent class.

The pixel distribution of the competition data and HPAv18 data are different from each other. This can degrade the generalization performance of the model. To address this, the input images were normalized by the mean and standard deviation of each image.

The input images are high-resolution and the objects in the image are small, so downscaling can cause loss of important information. On the other hand, even if high-resolution images are used, global average pooling can dilute the important information. The spatial attention method was used as a method to maintain important information while using high resolution images. To do so, the global average pooling layer was replaced by the following steps:

a) Compute $M \times M \times 28$ logits from last convolution layer using 1×1 convolution filter.
b) Compute $M \times M \times 28$ attention-map from last convolution layer using 1×1 convolution, followed by channel-wise softmax.
c) Element-wise multiplication with logits and attention-map.
d) Channel-wise sum

Additionally, to find an effective augmentation method, AutoAugment was used[9]. For simplicity, random searching was used.

For training the model, an Adam optimizer with a fixed learning rate 0.0005 was used. The final checkpoint was made using an average of the last 10 checkpoints from the epoch with the best F1 score. The output was thresholded using the ratio of labels in the training set[13].

Ablation Study

Experiment numbers in this section refers to indices from **Supplementary Table 5f**. A baseline model was trained with the Kaggle training dataset and 512×512 images. After adding HPAv18 data, the performance increased significantly (experiment #2). Experiment #3 verified that choosing thresholds for each label separately can overfit to the validation set. Changing augmentation, proportion based thresholding, and per-image normalization increased the performance slightly (experiment #4, #5 and #6). Finally, experiment #7 verified that large image sizes are important for performance.

After that, the performance of larger models was checked (experiments #8 and #9). Both of these models increased the performance on the public leaderboard significantly but only one of them, experiment #8, increased the performance on the private leaderboard.

Conclusions

For deep learning, it is crucial to have enough data. In this competition, the HPAv18 dataset played an important role in improving the performance of the model. One of the major challenges was also that rare classes did not have enough data. Finding an effective augmentation method was an important task in this challenge because the HPA images were very different from natural images like ImageNet data. AutoAugment made it possible to do this task without manual tuning. Instead of random searching, more effective methods like reinforcement learning could be used. With such methods, one can expect better performance out of the model.

4. Team 4 (Wienerschnitzelgemeinschaft) model summary

Method Overview

For this challenge, an ensemble of diverse and highly optimized CNN models were developed. Top performing CNN architectures include AirNet, AirNeXT, PreResNet, SE-ResNeXt, CBAM-ResNet, GAPNet, ResNet34, ResNet50xt, and Inceptionv3[14,15,16,18,]. While some of these models use attention mechanisms to deal with scale invariance and localization (e.g., AirNet, AirNeXT, CBAM-ResNet), other models (e.g., GAPNet) pool the output of different parts of a CNN model to account for different cell sizes.

The final two ensemble models can be compared in terms of trainable parameters used to compare model complexity. While the first model (**Supplementary Table 5g**, Final Ensemble1) was a relatively simple ensemble (0.498 billion parameters) of the best performing CNN architectures with experimentally optimal training and prediction configurations, the second (**Supplementary Table 5g**, Final Ensemble2) was a large ensemble (12.428 billion parameters) of multiple approaches and CNN architectures.

In the Final Ensemble1, weighted BCE was used as a loss function with a positive weight of 10 to deal with the extreme class imbalance. For most of the models in this ensemble, two versions were trained. The first version used an input image scaled to 512×512 px (RGB). The second version used the weights of the first version as pretrained weights, but took 512×512 px random crops of a 1024×1024 px (RGB) image as input. By using this technique, the model could be trained on larger images with the same architecture for a relatively shorter period of time. Various augmentation techniques were used to make the models robust: flipping, rotation, brightness, scaling, shearing, translation, and normalization. Normalization helped efficiently include the external HPAv18 dataset. Most of the models were trained for only 20 epochs. To ensemble the models, a label-specific cross-validated hill climber was applied[13]. Moreover, these models used pseudo-labeled data (data without ground truth labeled by the ensemble model) from the test dataset for training and it seemed to be helping in overall performance as the models see more data in total than in competition and HPAv18 data combined. We used a 2-fold cross validation strategy for this ensemble model.

In contrast, models for Final Ensemble2 came from different solution approaches. We tested GAPNet-like CNN architectures using feature maps from intermediate layers of a CNN. We also experimented with specifically designed loss functions to enable more common architectures like a simple ResNet34 to account for multiscale input and the localization task. The main idea was to downscale the green channel to a specific resolution (e.g., 32×32 px) and together with the image labels use it as a binary prediction map which had the shape $32 \times 32 \times 28$ due to the 28 classes. The segmentation loss of the prediction map was then used solely or in a weighted combination with the normal classification loss. We found these approaches (**Supplementary Table 5g**, models #9 and #10) performed comparably with standard multi-label classification approach. Stochastic Weight Averaging (SWA) of the models was also attempted in an attempt to further improve generalizability[16]. In general, SWA was helpful for model performance (**Supplementary Table 5g**, model #8). To increase variability to the approach, the training data was split based on different methods, such as 4-fold clustering of images, 5-fold stratified split, and 4-fold split using embedding features of training images from a ResNet18 model. This helped evaluate the models from different perspectives of a real-world scenario.

To ensemble the various solutions from different approaches in Final Ensemble2, weighted voting based on public leaderboard feedback was implemented and produced a comparable result to the hillclimber. A two-level stacking approach for the ensemble was also tested (**Supplementary Table 5g**, models #3 and #6). Stacking resulted in an insignificant boost to the score. Finally, the ensemble solution produced pseudo-labeled test data.

Ablation study

Experiment numbers in this section refers to indices from **Supplementary Table 5h**.

Image resolution. The experiments indicated that using higher resolution images, 512 × 512, improved the prediction performance significantly compared to 256 × 256 (**Supplementary Table 5h**: #1, #2). A similar trend could be seen while using even higher resolution images (1024 × 1024, Supplementary Table 5g: #1).

Image channel importance. As classification was dependant on the green channel, we experimented with assigning a greater weight to that channel. This did not improve the results (**Supplementary Table 5h**: #2, #10). However, removing the yellow channel yielded some performance gain (**Supplementary Table 5h** #16, #20). The yellow channel was kept for some models, while others were trained on RGB images.

Improvement within CNN architecture. In general, stochastic weight averaging (SWA) seems to improve model performance (**Supplementary Table 5h**: #4, #7). Modifying the classification head was also important to get good results (**Supplementary Table 5h**: #21).

Validation strategy. The validation strategy was crucial for training stable models. Image clustering based 4-fold and random stratified 5-fold resulted about similar performance (**Supplementary Table 5h**: #13, #14, #25, and #26). Though using different validation schemas did not improve individual model performance, it added some variety to the models. Moreover, we could analyze different aspects of overfit probability of the models by comparing between different strategies.

Adding external HPAv18 data. We experimented with adding external HPAv18 data to the competition data in different ratios. Both 2:1 and 1:2 ratio performed similarly (**Supplementary Table 5h**: #27, #28).

Threshold optimization for classification. Threshold optimization had a major impact in this challenge. We experimented with optimizing different class thresholds individually by approximately matching either the train set distribution, validation set distribution, or by best submission distribution. Surprisingly, optimizing the threshold by best submission (~0.640 public LB) distribution improved the previous score considerably (0.578 - 0.602 public LB; **Supplementary Table 5h**: #14, #15). Though the model capability was the same, a better score was achieved by searching the optimal threshold for the F1 metric.

Pseudo-label. Searching the optimal threshold iteratively and adding pseudo-label data in training improved the score to 0.620 public LB for a single CNN architecture (PreResNet50) at 512 × 512 resolution images (**Supplementary Table 5h**: #24). Further, ensembling from 6 different CNN architectures at 2 different resolutions (512 × 512 and 1024 × 1024) made the final ensemble score 0.653/0.568 on the public/private leaderboards, placing 4th on the private leaderboard.

Conclusions

In this competition, we have experimented with many proven techniques to improve the generalization ability of machine learning models. Some of the methods include robust validation

strategy, building strong single models, ensemble from diversified approaches, using as much data as possible and many others. In general, medium-sized CNN architectures (i.e., resnet34, resnet50) outperformed larger models (i.e., inceptionresnetV2, inceptionV3, wide-resnet). Using multi-scale training images (512 $\times$ 512 and 1024 $\times$ 1024) improved the prediction capability of models. Multi-scale training works well for most of deep learning methods. However, in this competition, some protein structures were really small to be classified by a 512 $\times$ 512 image and thus higher resolution helped. We have observed that adding more data to training by including HPAv18 dataset and pseudo label test dataset greatly improved model performance. However, adding extra data to training should be done in a careful way so that they contribute as little noise as possible (i.e., normalizing HPAv18 data, include a balanced portion of pseudo label data). Moreover, a robust cross-validation strategy helped us reducing overfit in training (i.e., 3 different validation fold construction strategies). Finally, understanding the competition metric was crucial to maintain a good rank. Some of the rare classes were highly impactful in the score even if using the same models. We have adopted an approach to approximately match our best submission class distribution in the next submission, which helped us in finding the optimal threshold for classification.

5. Team 5 (VPP) model summary

Method Overview

The base networks of the model were Inception-v3, Inception-v4, and Xception, all pre-trained on ImageNet[6,19,20]. For the competition classification task, the following modifications were made:

1. Changed the number of input channels of first convolution to 4 (red, green, blue, yellow).
2. Changed the last pooling layer to global average pooling.
3. Appended a fully connected layer with output dimension 128 after the global pooling.
4. Appended a batch normalization layer and a dropout layer before the additional fully connected layer.

We used both official (both PNG and TIFF) datasets and the HPAv18 external data. Three different scales were used during training (512 × 512 for PNG images and 650 × 650, 800×800 for TIFF images). The HPA dataset has four channels each of which is an RGB image of its own, so we took only one channel (r=Hrr, g=Hgg, b=Hbb, y=Hyb, where Hyb is the blue channel of the yellow dyeing mode image from HPAv18 dataset) to form a 4-channel (r,g,b,y) input for training. Rotation, flip, and shear were very effective augmentations to increase the amount of training data.

To handle the class imbalance, different sampling weights were set for different classes, to ensure that categories with fewer labels have a higher probability of sampling. A multi-label one-versus-all loss based on max-entropy (MultiLabelSoftMarginLoss) was used for all the models across 10-fold cross validation sets with 8% for validation. All the models were trained with stochastic gradient descent (SGD) with momentum set to 0.9, and weight decay of 1e-4. The initial learning rate for input size 512 × 512 was set to 0.05, and 0.01 for 650 × 650 and 800 × 800. A step learning rate scheduler with gamma of 0.1 and step of 6 was applied. The training process was divided into two stages, where the first stage used 512 × 512 with models trained on ImageNet, and the second stage used 650 × 650 or 800 × 800 with model trained from the first stage.

The features of Inceptionv3, Inceptionv4 and Xception with 10 folds and size 800 × 800 were extracted, and then concatenated into a new feature vector with 3840 dimensions. A sample multi-layer perceptron network was created to classify 28 categories from the new features, with three fully connected layers (3840, 512, and 28 neurons respectively). Two loss functions were tested: MultiLabelSoftMarginLoss and BCEWithLogitsLoss, which reached scores of 0.5515/0.62791 and 0.55227/0.62963 respectively.

The final model is an ensemble of the above methods as described in **Supplementary Table 5i**.

Ablation Study

Experiment numbers in this section refers to indices from **Supplementary Table 5j**.

Initially, a number of base CNN networks such as VGG, ResNet, Inception, Xception, DenseNet, DPN, SE-ResNet were trained, using BCEWithLogitsLoss on the official training data with input size 512 × 512 × 4. Each network was trained for about 15 epochs. The results of these experiments (#7, #11, #13, and #15), show that DenseNet and Inception models perform better than the others.

DenseNet-121 was initialized the network with the models pre-trained on the ImageNet dataset,trained on the extra HPAv18, and then fine-tuned with the official data, experiment #1 and #2. No noticeable improvement was made, so the network was modified by adding another fully

connected layer, experiment #3, and incorporated weighted sampling techniques, experiment #4. Each method increased the score by about 0.02 on its own and, after combining the methods, the score was boosted by about 0.035 in experiment #5. Then we combine HPAv18 data and the official data together to train DenseNet-121 directly. This strategy achieved 0.532 on the public leaderboard, experiment #6. In particular, this increased the score by about 0.1 on Inception-v3 (#13), v4 (#15), and Xception (#11).

Another strategy that was used to improve performance was the multi-size scheme. In practice, 512, 650, and 800 were used as input sizes, where 512 was used in the pre-training/warm-up stage, followed by fine-tuning with 650 and 800 (**Supplementary Table 5i**). Slightly better performance was achieved with 800 (experiment #7 and #11) instead of 650, achieving at least 0.58 in the second stage on the leaderboard for all three backbone networks (Inception-v3, -v4, and Xception) that was tested.

Finally, we used retrieval method for label-switching. In detail, we used the 512-D features produced by Inception-v4 and took, for each test image, obtained its closest neighbor in the HPAv18 dataset using cosine similarity, and took the given labels of the matched image for the test image. We did this for about 300 test images that had the highest confidence from the previous prediction. This method increased the score by about 0.03-0.05 on the public leaderboard, but unfortunately it brought the private score down.

Conclusions

Based on the experiments, the choice of a backbone network is crucial. For instance, in this particular task, DenseNet and Inception performed much better than other popular networks such as ResNet. To deal with class imbalance, weighted sampling helped a lot.Multi-size and Test Time Augmentation also appeared to be beneficial.

6. Team 8 (One More Layer of Stacking) model summary

Method Overview

In addition to the challenges of multi-label classification and the high class imbalance, the dataset had several features that sets it apart from prior image classification tasks. For example, the regions of interest were small and could be located anywhere in the image. The data was also quite different from usual ImageNet images, so transfer learning should have been applied carefully.

The solution consisted of fourteen neural networks aggregated to an ensemble via stacking (**Supplementary Fig. 10**). The different architectures involved in the ensemble were se-resnext-50, inception-v4, xception and bn-inception[6,]. All networks were trained with brightness augmentations, rotations, zoom and crop, D4 symmetry group and wrap transforms. For prediction, the same augmentations were used and averaged 32 Test Time Augmentation(TTA) predictions. Then, we calculated the final prediction as 0.4*original + 0.6*TTA.

For all models, the subsequent fine-tuning strategy was used with small variations:

1. Train only networks head with high LR (1e-3 usually). The best strategy was to train networks head to convergence.
2. Unfreeze other layers. Train them with cyclic learning rate. LR finder was used to select upper bound of a cycle for cyclic learning rate. Lower bound was 25 times smaller than the upper bound.
3. Differential learning rate was applied to allow effective transfer learning taking into account that the nature of data was quite different from that of ImageNet on which the networks were pretrained. The learning rate for the first layer is 16 times lower than for the last one. Intermediate layers had intermediate learning rate values distributed logarithmically from the lower bound to the upper bound.

Wadam was used as an optimizer. Focal loss (gamma=2) and LSEP loss were used as an optimization objective to handle class imbalance[10,21]. The networks were trained for 2-4 cycles. Usually for 16 epochs during early cycles of training and 8 epochs closer to the end. Almost all models (except #13) were trained on RGB channels, since the endoplasmic reticulum channel did not show performance improvement.

Training on high resolution (more than 1024 $\times$ 1024) did not work at all. Simple training on crops from the full size image did not work either, since there are classes located in a single point of an image (e.g., cytokinetic bridge).

Our team used HPAv18 data in addition to the competition dataset for training. For validation purposes, an Xception network was trained on the train and test data to classify whether the sample was from train or from test (adversarial validation). This classifier had 0.6 ROC-AUC, which meant that training data differs from the data provided as part of the competition. This could be explained by the pixel distribution differences between HPAv18 and the test data. After that we selected 25% of train data which is the closest to the test dataset as our validation holdout for further hyperparameters tuning and architecture selection.

Our models were stacked with LightGBM on this holdout. We used probabilistic predictions of all networks for each class and some meta-features (e.g. image brightness) for stacking.Stacking was implemented in a folds-in-folds manner with voting of several averaged iterations of stacking to

eliminate the impact of selecting threshold for f1-score. We applied class weights and oversampling during stacking.

Ablation study

The experiment numbers in this section refers to indices from **Supplementary Table 5I**.

Our team tested several other architectures than the one described above (DenseNet-201 and ResNet-34, #3 and #4), but they showed lower f1-score on the cross-validation.

Also, changing input shape from 512x512 to 256x256 and 768x768 was tested (#7, #8, and #9). It showed that with lower input size models underfitted while with higher input size they overfitted.

Conclusions

Based on our experiments, SE-ResNext and Inception-based architectures performed reasonably well on this task.

Dealing with the class imbalance was crucial - to handle it we used focal loss and oversampling with class weights during ensembling.

Image input size also mattered. Training on higher input size tended to overfitting and required more hardware to maintain required batch size. Training on lower resolution did not provide acceptable results. The results indicate that an input size of 512x512 is the optimal image size for this model.

7. Team 10 (Conv is all you need) model summary

Method Overview

The final model is an ensemble of three CNN models (**Supplementary Fig. 11a** and **Supplementary Table 5m**). Of these, the best performing single-model (achieving private LB score of 0.54 without late submission) during the competition was an Xception-based model on target-based cropping windowed images (CWXception)[6]. The other two CNN models are SE-ResNext50 and InceptionV3, respectively[5,22].

For CWXception, the original image was resized into 960 × 960px, then cropped into ten 384×384 windows (**Supplementary Fig. 11b**) based on green channel signals. These were then fed into an ImageNet (http://www.image-net.org/) pre-trained Xception network. The class-balanced sampling helped the model quickly converge to a training optimal compared to traditional batch training. Randomly target (green)-removal in batches (and setting as negative labels) helped the model better focus on the essential targets instead of remembering reference channels.

Additionally, we built an interactive graphical user interface (GUI, **Supplementary Fig. 11c**, https://i.imgur.com/OJ4NA9r.gifv) to superimpose the model prediction scores with corresponding localized windows onto the original image, thus encouraged us a better understanding of our model forecasting and facilitated human sanity check efficiently. The cropping window provides another way of data augmentation and prediction ensembling, thus to improve the model performance.

Both SE-ResNext50 and InceptionV3 models were pre-trained on ImageNet and trained on the whole 512 × 512 images. To capture information from a low-level encoder, we added auxiliary supervision branches after several intermediate layers[23]. The deeply supervised structure accelerated training efficiency and improved prediction performance.

Ablation study

The experiment numbers in this section refers to method indices from **Supplementary Table 5n**.

For both vanilla SE-ResNext50 and InceptionV3 which were trained on 512 × 512 4-channel images with external data, we found deep supervision increased models' performances when we compared #2 to #1 and #7 to #6. Five-fold ensemble could significantly improve the LB score (#3 vs #4). Other techniques, e.g., test time augmentation, different thresholding, additionally helped with the increment of model classification (#4, #5 vs #3).

For the CWXception, we observed significantly augmentation of LB scores by using cropping window technique tandem with class balanced sampler (#9 vs #8). Although Xception demonstrated slightly lower LB scores than InceptionV3, it contains fewer trainable-parameters. We thus applied Xception as our base model. Similarly, five-fold ensemble and threshold tuning improved our model's prediction performance (#11, #12 vs #10). In addition, we further trained the model with non-target (green) regularization and achieved slightly improved LB scores.

Conclusions

Our models nicely combined two very different approaches, global models which were responsible for capturing the global configuration of the image, and localized (window-cropped) model which was responsible for discerning the minute details in the image. We observed that the two approaches perform very differently in terms of their per-class f1, which indicates that the two types of approaches synergize very well, filling in each other's weaknesses.

For the localized model, we developed a GUI tool to visualize the localization and prediction of cropping windows that played an essential role in our model iterations. By having all the relevant information displayed on the same console, we were able to quickly identify weaknesses and errors in our model/training and propose improvement measures in the next iteration. This "fast iteration" approach is one of our best advice for building good models.

8. Team 16 (NTU_MiRA) model summary

Method Overview

Training CNN with large input size (1024 × 1024 px) using fixed batch-normalization and data distillation.

Resnet34 was used as the backbone network, replacing the head of the network with 1 fully-connected layer with 28 hidden units. The learning rate was chosen by LR range test, as proposed by, to find original learning rate[26]. The max number of epochs and decay rate were tuned according to results from the public leaderboard.

In the beginning, only the newly added fully-connected layer was trained with a learning rate 1e-3 for 4 epochs. After that, the whole network was trained with 1e-4 and decay by a factor of 0.1 from epoch 15 until epoch 25. The final threshold for prediction was fixed at 0.15 based on public LB score.

Resnet34 was trained using 1024 × 1024 px images on 2 P100 gpus (batch size=8). Training for one epoch took around 45 minutes, with gpu utility around 85%.

Gradient Accumulation. To overcome the small batch size we forward 4 times then updated the weights in order to maintain effective batch size to 32.

Freeze batch-normalization Layer. Batch normalization could cause negative impact when training with small batch size[8]. Simply removing batch normalization layer will greatly impact the imagenet pretrained model. As a result, the batch normalization parameters were frozen both during training and testing.

Ablation Study

Experiment numbers in this section refers to indices from **Supplementary Table 5p**.

The baseline result using resnet34, experiment #1, with one fully connected layer shows that rare labels are not well learned, since the labels are highly imbalanced). In experiment #2, log scaled median-frequency balancing was used to calculate the weight[24]. These weights (rounded to the nearest integer and tuned based on public leaderboard) were used to oversample only the rare labels. To further alleviate the imbalance problem the external HPAv18 data was added in experiment #3 and the score was improved significantly.

As described by Rumetshofer et. al, the resolution has great impact on performance[15]. However, when trying to fit the 1024 × 1024 images into p100 GPUs it was only possible with a batch size of 2. As the weights were pretrained from ImageNet, batch normalization actually bring the score down. To combat the issue, the parameters of batch-normalization layers were frozen.

Data distillation was applied to fight the label imbalance issues[25]. We used a 5-fold model with all test-time-augmentation to predict on testing data, experiment #5 and #7, which increased the performance significantly. The Public/Private LB score is 0.613/0.537 (using 512 × 512) and 0.620/0.542 (using 1024 × 1024).

Using the prediction as ground truth and perform the same training process we get 0.634/0.549, experiment #8.

The final submission only used the second stage model to avoid overfitting on public LB and performed simple post-processing (remove prediction more than 6) to get 0.629/0.553, experiment #10.

Conclusions

The model is pretty standard, and only pretraining the fully connected layer at first was crucial. During the competition, overfitting was the biggest problem. Therefore, carefully selecting the correct sampling strategy and validation set was very important.

The biggest disadvantage of this solution is that the protein location information is not used. There was not enough time to leverage other channel information, especially what is available in the yellow channel.

9. Team 39 (Random Walk) model summary

Method Overview

Our final model was an averaged ensemble of same CNN models with three different resolutions (**Supplementary Fig. 13a**). Resnet18 was used as the backbone, and the feature maps of the last three blocks were used to generate soft-attention and self-gating[27]. The self-attention mechanism generates a gating signal that is end-to-end trainable, which allows the network to contextualise local information useful for prediction. The attended features at last 3 blocks of Resnet18 are combined for a final prediction by aggregating the mean. The original images (512 $\times$ 512) were cropped into 3 different sizes (256 $\times$ 256, 384 $\times$ 384, and 512 $\times$ 512) to fit 3 separate AGN models, before finally ensembling them. The solution only used a single model and 512 $\times$ 512 PNG files with HPAv18 external data, without any use of cross validation.

For a chosen feature map based on the last feature map of block2 or block3, the global features were used to joint generate attention score maps for the relevant information regarding the protein of interest. First, two 1 $\times$ 1 convolutions projected the chosen feature map and global feature map into the same dimension (**Supplementary Fig. 13b**). The global feature map was upsampled to the same resolution as chosen feature map. The two feature maps were added and processed by another 1 $\times$ 1 convolution to generate an attention score map. Finally, the chosen feature map was multiplied by the attention score map to generate the aggregated feature map.

Our model was optimized by SGD with a momentum of 0.9. Training started with a learning rate of 0.1 and reduced by factor 0.2 when the loss stop decreased in 8 epochs. The images are augmented through random cropping and random flipping. We trained our model to optimize the loss function which is the sum of focal loss and soft F1 loss with weight 1:1.

Ablation Study

The experiment numbers in this section refers to method indices in **Supplementary Table 5q**.

We tested our model conditioned on different backbone and different loss combination. We experimented on three popular backbones (VGG, Inception V3, Resnet) and three losses (soft F1 loss, focal loss, BCE loss). Experiments #1 to #3 studied the influence of different backbones, and the results showed that resnet works better than VGG or inception V3 in the similar parameter size level. For experiments # 4 to #6, we studied different loss functions and resample strategy. In our results, soft F1 loss was vital for better public/private LB, while focal loss and weighted BCE loss contributed nearly the same to our model performance. We used batch size 256, so soft F1 loss were relatively stable during our training process cause we chose smaller backbones (resnet18, vgg13_bn) and a smaller resolution (256 x 256 px). Experiment #4 showed that resample strategies do not work for our model.

Conclusions

This task is particularly difficult because the pattern of the protein of interest is small compared to the size of the input image and because the background of the images are similar to each other. For a standard recognition model, global average pooling can aggregate global information for classification, but it loses local information. We used the global feature of the last feature map before global average pooling to jointly attend the different scale CNN level features and ensemble the different scale prediction score which benefit the prediction performance.

References

1. Masi, I., Wu, Y., Hassner, T., & Natarajan, P. . Deep face recognition: A survey. *Conference on graphics, patterns and images,* 2018b.
2. J. Deng, J. Guo, and S. Zafeiriou, ArcFace: Additive Angular Margin Loss for Deep Face Recognition, *arXiv preprint arXiv:1801.07698,* 2018.
3. Huang G, Liu Z, Van Der Maaten L, et al. Densely connected convolutional networks[C] *IEEE Conference on Computer Vision and Pattern Recognition (CVPR). IEEE, 2261-2269,* 2017.
4. Pan X, Luo P, Shi J, et al. Two at once: Enhancing learning and generalization capacities via ibn-net[C],*Proceedings of the European Conference on Computer Vision (ECCV).: 464-479*, 2018.
5. Hu J, Shen L, Sun G. Squeeze-and-excitation networks[J]. *arXiv preprint arXiv:1709.01507*, 2017.
6. Chollet F. Xception: Deep learning with depthwise separable convolutions[J]. *arXiv preprint*,: *1610.02357,* 2017.
7. Kingma D P, Ba J. Adam: A method for stochastic optimization[J]. *arXiv preprint arXiv:1412.6980*, 2014.
8. Smith L N. Cyclical learning rates for training neural networks[C], *Applications of Computer Vision (WACV), 2017 IEEE Winter Conference on. IEEE,464-472,* 2017.
9. Ekin D Cubuk, Barret Zoph, Dandelion Mane, Vijay Vasudevan, and Quoc V Le. AutoAugment: Learning augmentation policies from data. *arXiv preprint arXiv:1805.09501*, 2018.
10. T.-Y. Lin, P. Goyal, R. Girshick, K. He, and P. Dollar. Focal loss for dense object detection. *arXiv preprint arXiv:1708.02002*, 2017
11. C. Szegedy, V. Vanhoucke, S. Ioffe, J. Shlens, and Z. Wojna. Rethinking the inception architecture for computer vision. *Proceedings of the IEEE conference on computer vision and pattern recognition*. 2016
12. K. He, X. Zhang, S. Ren, and J. Sun. Deep residual learning for image recognition. *arXiv preprint arXiv:1512.03385*, 2015.
13. P. Izmailov, D. Podoprikhin, T. Garipov, D. Vetrov, and A. G. Wilson. Averaging weights leads to wider optima and better generalization. *Uncertainty in Artificial Intelligence (UAI)*, 2018.
14. R. Caruana, A. Munson, and A. Niculescu-Mizil. Getting the most out of ensemble selection. *sixth International Conference on Data Mining(ICDM'06), 828–833*, 2006.
15. Elisabeth Rumetshofer, Markus Hofmarcher, Clemens R ¨ohrl, Sepp Hochre-iter, and G ¨unter Klambauer. Human-level protein localization with convolutional neural networks. *International Conference on Learning Representations*, 2019.
16. Sanghyun Woo, Jongchan Park, Joon-Young Lee, and In So Kweon. CBAM: convolutional block attention module.*CoRR, abs/1807.06521*, 2018.

17. L. Yang, Q. Song, Y. Wu, and M. Hu. Attention inspiring receptive-fields network for learning invariant representations. *IEEE Transactions on Neural Networks and Learning Systems, 1–12,* 2018.

18. Kaiming He, Xiangyu Zhang, Shaoqing Ren, and Jian Sun. Identity mappings in deep residual networks. *CoRR, abs/1603.05027*, 2016

19. C.Szegedy,V.Vanhoucke,S.Ioffe,J.Shlens,andZ.Wojna. Rethinking the inception architecture for computer vision. *arXiv preprint arXiv:1512.00567*, 2015.

20. Szegedy C, Ioffe S, Vanhoucke V, et al. Inception-v4, inception-resnet and the impact of residual connections on learning[C]. *Thirty-First AAAI Conference on Artificial Intelligence,* 2017.

21. Yuncheng Li et al. Improving Pairwise Ranking for Multi-label Image Classification. *arXiv preprint arXiv 1704.03135v3,* 2017.

22. Szegedy, C. et al. Going deeper with convolutions. *Proceedings of the IEEE Computer Society Conference on Computer Vision and Pattern Recognition 7-12-NaN-2015, 1–9,* 2015.

23. Wang, L., Lee, C.-Y., Tu, Z. & Lazebnik, S. Training deeper convolutional networks with deep supervision. *arXiv preprint. ArXiv1505.02496*, 2015.

24. Eigen, David, and Rob Fergus. Predicting depth, surface normals and semantic labels with a common multi-scale convolutional architecture. *Proceedings of the IEEE International Conference on Computer Vision,* 2015.

25. Radosavovic, Ilija, et al. Data distillation: Towards omni-supervised learning. *2018 IEEE/CVF Conference on Computer Vision and Pattern Recognition. IEEE*, 2018.

26. Wu, Yuxin, and Kaiming He. Group normalization. *arXiv preprint arXiv:1803.08494,* 2018.

27. Schlemper J, Oktay O, Chen L, et al. Attention-gated networks for improving ultrasound scan plane detection[J]. *arXiv preprint arXiv:1804.05338*, 2018.
